# *yqhG* Contributes to Oxidative Stress Resistance and Virulence of Uropathogenic *Escherichia coli* and Identification of Other Genes Altering Expression of Type 1 Fimbriae

**DOI:** 10.3389/fcimb.2019.00312

**Published:** 2019-08-29

**Authors:** Hicham Bessaiah, Pravil Pokharel, Hajer Habouria, Sébastien Houle, Charles M. Dozois

**Affiliations:** ^1^INRS-Centre Armand-Frappier Santé Biotechnologie, Laval, QC, Canada; ^2^CRIPA-Centre de Recherche en Infectiologie Porcine et Avicole, Saint-Hyacinthe, QC, Canada

**Keywords:** *Escherichia coli*, urinary tract, type 1 fimbriae, luciferase, stress

## Abstract

Urinary tract infections (UTIs) are common bacterial infections and the vast majority of UTIs are caused by extraintestinal pathogenic *Escherichia coli* (ExPEC) strains referred to as uropathogenic *E. coli* (UPEC). Successful colonization of the human urinary tract by UPEC is mediated by secreted or surface exposed virulence factors—toxins, iron transport systems, and adhesins, such as type 1 fimbriae (pili). To identify factors involved in the expression of type 1 fimbriae, we constructed a chromosomal transcriptional reporter consisting of *lux* under the control of the fimbrial promoter region, *fimS* and this construct was inserted into the reference UPEC strain CFT073 genome at the *att*Tn7 site. This *fimS* reporter strain was used to generate a Tn*10* transposon mutant library, coupled with high-throughput sequencing to identify genes that affect the expression of type 1 fimbriae. Transposon insertion sites were linked to genes involved in protein fate and synthesis, energy metabolism, adherence, transcriptional regulation, and transport. We showed that YqhG, a predicted periplasmic protein, is one of the important mediators that contribute to the decreased expression of type 1 fimbriae in UPEC strain CFT073. The Δ*yqhG* mutant had reduced expression of type 1 fimbriae and a decreased capacity to colonize the murine urinary tract. Reduced expression of type 1 fimbriae correlated with an increased bias for orientation of the *fim* switch in the OFF position. Interestingly, the Δ*yqhG* mutant was more motile than the WT strain and was also significantly more sensitive to hydrogen peroxide. Taken together, loss of *yqhG* may decrease virulence in the urinary tract due to a decrease in production of type 1 fimbriae and a greater sensitivity to oxidative stress.

## Introduction

Urinary tract infections (UTIs) can occur throughout the urinary tract within the urethra, bladder, ureters, or kidneys. UTIs are a common infectious disease with over 150 million cases documented worldwide each year (Stamm and Norrby, 2001; Foxman, 2014). Furthermore, the overwhelming majority of uncomplicated UTI cases (≥80%) are caused by extraintestinal pathogenic *Escherichia coli* (ExPEC), referred to as uropathogenic *E. coli* (UPEC) (Ronald, 2002), which can belong to a diversity of phylogenetic groups or sequence types (Russo and Johnson, 2000; Chen et al., 2013). An estimated 50% of women experience at least one UTI during their life, and up to a quarter of those women are prone to recurrent UTIs (Foxman, 2002). Adherence of UPEC to host cells is a key event in initiating UTI pathogenesis and is important for overcoming strong urine flow, and to promote urinary tract colonization (Ulett et al., 2013; Flores-Mireles et al., 2015).

Fimbriae (pili) are filamentous structures that can mediate adherence of bacteria to host cell receptors (Mulvey et al., 1998; Nielubowicz and Mobley, 2010). For instance, type 1 fimbriae are critical for colonization of the bladder by UPEC (Gunther et al., 2002), to directly stimulate UPEC invasion into epithelial cells and aid in formation of intracellular reservoirs that may contribute to recurrent infection (Mysorekar and Hultgren, 2006). Type 1 fimbriae encoded by the *fimAICDFGH* (*fim*) genes, are one of the best-characterized UPEC chaperone-usher fimbriae. These fimbriae are produced by most *E. coli* strains including UPEC (Buchanan et al., 1985; Sivick and Mobley, 2010). The *fimA* gene encodes the type 1 major fimbrial subunit and the tip-located adhesin, encoded by *fimH*, mediates binding to α-d-mannosylated receptors, such as uroplakins, which are abundant in the bladder and other host surfaces containing mannosides (Connell et al., 1996; Wu et al., 1996). The *fim* promoter is located on a 314-bp invertible DNA promoter element (*fimS*), the orientation of which determines the transcriptional status (ON or OFF) (Abraham et al., 1985; Bjarke Olsen and Klemm, 1994). The switching orientation of *fimS* is controlled by two recombinases. FimB, which promotes inversion in both orientations, and FimE, which mediates the switching from phase-on to phase-off (Klemm, 1986; Gally et al., 1996). Two additional recombinases, IpuA and IpbA, present in UPEC strain CFT073 can also mediate switching of the invertible element independent of FimB and FimE (Bryan et al., 2006). Regulation of *fim* genes is affected by multiple environmental factors (including pH, osmolarity, temperature and oxygen levels) and at least three regulatory proteins are directly implicated (Lrp, IHF, and H-NS) (Unden and Kleefeld, 2004).

To establish a UTI, UPEC strains must resist environmental stresses in the bladder and kidneys including pH stress and wide fluctuations in osmolarity (Culham et al., 2001; Cahill et al., 2003). The high osmolality, high urea concentration, acidic pH and organic acids in urine can limit the growth and survival of *E. coli* within the urinary tract (Mulvey et al., 2000). Thus, an important aspect of UPEC virulence is the capacity to resist high osmolality and the denaturing effects of urea, and rapidly adapt to changes and stress encountered in these niches during establishment of the infection.

Previously, we showed that interference with phosphate homeostasis decreased the expression of type 1 fimbriae in strain CFT073 and attenuated UPEC virulence (Crépin et al., 2012b). The phosphate-specific transport (Pst) system negatively regulates the activity of the two-component signal transduction system PhoBR and also transports inorganic phosphate. Inactivation of the Pst system results in constitutive activation of PhoBR regardless of environmental phosphate availability (Wanner, 1996). As such, a *pst* mutant responds as if it is always under phosphate-limiting conditions. In UPEC, we showed that decreased virulence of a *pst* mutant is largely due to reduced expression of type 1 fimbriae. In avian pathogenic *E. coli* (APEC), an altered membrane homeostasis was also observed in a *pst* mutant (Lamarche and Harel, 2010) and caused increased sensitivity to acid, cationic antimicrobial peptides, and serum (Lamarche et al., 2005; Crépin et al., 2008; Bertrand et al., 2010).

In order to identify genes affecting the expression of type 1 fimbriae, we constructed a transcriptional luciferase (*lux*) reporter consisting of *lux* under the control of the *fimS* invertible promoter and this construct was inserted into the CFT073 genome at the *att*Tn7 site. This *fimS* reporter containing strain was used to generate a Tn*10* transposon mutant library, coupled with high-throughput sequencing to identify the location of transposon insertions that altered the expression of type 1 fimbriae. In this report, we show that YqhG is one of the important mediators that contribute to decreased expression of type 1 fimbriae in UPEC strain CFT073. Our results demonstrated that the deletion of *yqhG* in CFT073 reduced the expression of type 1 fimbriae and reduced urinary tract colonization of the *yqhG* mutant in the murine model. We also examined whether deletion of *yqhG* as well as the *pst* system also reduced resistance to environmental stresses, suggesting that altered expression of type 1 fimbriae can be linked to changes in bacterial adaptation to environmental stresses, such as oxidative or osmotic stress.

## Materials and Methods

### Bacterial Strains, Growth Conditions, and Plasmids

*E. coli* strains and plasmids used in this study are listed in Table 1. *E. coli* CFT073 was isolated from the blood and urine of a patient with acute pyelonephritis (Mobley et al., 1990). Bacteria were routinely grown in lysogeny broth (LB) (Alpha Bioscience, Baltimore, MD) at 37°C and in human urine. Urine was obtained from healthy female volunteers, 20–40 years old, with no occurrence of a UTI or antibiotic use within the last 2 months prior to collection. A protocol for obtaining biological samples from human donors was reviewed and approved by the ethics committee—Comité d'éthique en recherche (CER 19-507) of INRS. Urine was immediately filter sterilized (0.2-μm pore size), pooled, and frozen at −80°C and used within 2 weeks of sampling. Antibiotics and reagents when required were added at the following concentrations: kanamycin, 50 μg/ml; ampicillin, 100 μg/ml; chloramphenicol 30 μg/ml and diaminopimelic acid (DAP), 50 μg/ml.

**Table 1:** Bacterial strains and plasmids used in this study.

**Strain or plasmid**	**Relevant characteristics**	**Reference or source**
**STRAINS**		
CFT073	UPEC wild-type pyelonephritis strain (O6:K2:H1)	Mobley et al., 1990; Welch et al., 2002
QT1324	CFT073 Δ*oxyR*::Km; Km^r^	Crépin et al., 2012b
QT1911	CFT073 *ΔpstSCA::FRT*	Crépin et al., 2012b
QT2087	MGN-617 + pLOF/Km; Ap^r^, Km^r^	Crépin et al., 2017
QT2117	QT1911::Tn*7*T-Gm::*pstSCA*; Gm^r^	Crépin et al., 2012b
QT2138	CFT073 Δ*fimAICDFGH*::km; Km^r^	Crépin et al., 2012b
QT2496	CFT073 + pSTNSK, Km^r^	Crépin et al., 2012a
QT4791	χ7213 + pGP-Tn7-Cm-*PfimA* L-ON *luxCDABE*, Ap^r^, Cm^r^, Km^r^	This study
QT4792	χ7213 + pGP-Tn7-Cm-*PfimA* L-ON *luxCDABE*, Ap^r^, Cm^r^, Km^r^	This study
QT4793	χ7213 + pGP-Tn7-Cm-*PfimA* phase variable *luxCDABE*, Ap^r^, Cm^r^, Km^r^	This study
QT4794	QT2496::Tn7T-Cm::*PfimA L-ON luxCDABE*, Cm^r^	This study
QT4795	QT2496::Tn7T-Cm::*PfimA-L-OFF luxCDABE*, Cm^r^	This study
QT4796	QT2496::Tn7T-Cm::*PfimA* phase variable *luxCDABE*, Cm^r^	This study
QT4976	CFT073 *ΔpstSCA*::FRT +pSTNSK, Km^r^	This study
QT5018	QT4976::Tn7T-Cm- *PfimA* phase variable *luxCDABE*, Cm^r^	This study
QT5134 (BW25123)	*E. coli* BW25123, *yqhG::Km*	Baba et al., 2006
QT5178	CFT073 *ΔyqhG*::FRT	This study
QT5235	QT5178::Tn7T-Cm::*yqhGH*, Cm^r^	This study
χ7213 (MGN-617)	*thi thr leu tonA lacY glnV supE ΔasdA4 recA*::RP4 2-Tc::Mu [λpir], Km^r^	Kaniga et al., 1998
**PLASMIDS**		
pCP20	FLP helper plasmid Ts replicon; Ap^r^ Cm^r^	Datsenko and Wanner, 2000
pGP-Tn7-Cm	pGP-Tn7-FRT::Cm, Ap^r^, Cm^r^	Crépin et al., 2012a
pSTNSK-	pST76-K::*tnsABCD*, Km^r^	Crépin et al., 2012a
pIJ461	pGP-Tn7-Cm::*luxCDABE;* Ap^r^, Cm^r^	This study
pIJ514	pGP-Tn7-Cm::*luxCDABE*; *rbs* Ap^r^, Cm^r^	This study
pIJ516	pGP-Tn7-Cm::*PfimA* L-ON *luxCDABE*, Ap^r^, Cm^r^	This study
pIJ517	pGP-Tn7-Cm::*PfimA-*L-OFF *luxCDABE*, Ap^r^, Cm^r^	This study
pIJ518	pGP-Tn7-Cm::*PfimA* phase variable *luxCDABE*, Ap^r^, Cm^r^	This study
pIJ543	pGP-Tn7-Cm::*yqhGH*, Ap^r^, Cm^r^	This study
pKD3	Template plasmid for the amplification of the *cat* gene bordered by FRT sites	Datsenko and Wanner, 2000
pKD4	Template plasmid for the amplification of the *km* cassette bordered by FRT sites	Datsenko and Wanner, 2000
pKD46	λ-Red recombinase plasmid Ts replicon; Ap^r^	Datsenko and Wanner, 2000
pLOF/km	Tn*10*-based transposon vector delivery plasmid; Ap^r^ Km^r^	Herrero et al., 1990

### Construction of the *fim-lux* Reporter Fusions

*E. coli* CFT073 harboring the *fimS* reporter was obtained by site-specific transposition of the *fimS-lux* genes at the chromosomal *att*Tn*7* site as described by Crépin et al. (2012a). Briefly, the promoterless *lux* operon of *Photorhabdus luminescens* (*luxCDABE*) (Allen, 2000) was amplified with the primers CMD1733 and CMD1734 (Supplemental Table 1). This DNA fragment was digested with KpnI and ApaI (New England Biolabs), purified with the Biobasic kit and ligated into the multiple-cloning sites (MCS) of the mini-Tn*7*-containing vector pGP-Tn7-Cm, generating the vector pGP-Tn7-*lux* (pIJ461). An optimized ribosome-binding site, RBS, was then added to plasmid pIJ461 to generate the pIJ514 vector. Then, the plasmid pIJ514 was used to generate the vectors, pIJ516 *pfimA* phase L-ON, pIJ517 pfimA phase L-OFF and pIJ518 *pfimA* variable phase. The *fim* promoter from strain CFT073 was amplified by PCR with primers CMD1645 and CMD1646 (see Supplemental Table 1). Genomic DNA, digested with the restriction enzymes *Eco*RI and SalI, and ligated to pIJ514 previously digested with EcoRI and *Xho*I. Transformation into *E. coli* DH5α cells was followed by selection on LB plates containing chloramphenicol. Using the approach described by Gunther et al. (2002), point mutations in primer CMD1133 were introduced to block the promoter switch in the ON position, digested with the restriction enzymes SmaI and SalI, and ligated to pIJ514 plasmid digested with SmaI and XhoI. The resulting vectors pIJ516, pIJ517, pIJ518 were transformed in *E. coli* SM10 λ*pir*-derivative strain MGN-617.

Strain MGN-617 (pGP-Tn7-*fimS-lux*) was conjugated overnight with strain CFT073, containing plasmid pSTNSK, which encodes the Tn*7 tnsABCD* transposase genes, at 30°C on LB agar plates supplemented with DAP. Following overnight culture, the bacteria from agar plates were suspended in 1 ml of phosphate-buffered saline (PBS), washed twice in PBS, serially diluted, and cultured on LB agar supplemented with gentamicin, and incubated at 37°C. Colonies that grew were then tested for sensitivity to kanamycin and ampicillin, indicating the likelihood of integration at *att*Tn7 and loss of the transposase-encoding plasmid pSTNSK. Insertion of Tn*7* into the *att*Tn7 site was verified by PCR (primers CMD26 and CMD1416 (see Supplemental Table 1).

### Testing the *fim-lux* Fusions Under Different pH Conditions

To measure the changes following growth in media at different pH, LB was buffered using 0.1 M Na_2_HPO_4_-NaH_2_PO_4_ buffer. The media were prepared with a pH ranging between 4.4 and 7.0. *E. coli* containing *pfimA phase-variable, pfimA L-ON* and *pfimA-L-OFF lux* fusions were incubated overnight at 37°C, 250 rpm in 5 ml LB medium. The next day 3 μl of each overnight culture was transferred to 180 μL of buffered LB at a specific pH and incubated with agitation until mid-logarithmic phase had been reached in 96-well plates at 37°C. The luminescence and O.D._600nm_ was measured each 15 min for 4 h using a Cytation™ 3 Cell Imaging Multi-Mode Reader (BioTek Instruments Inc.). The luminescence results were reported as relative luminescence units (RLU). The luminescence readings were normalized to the O.D._600nm_ values. The luminescence of *lux* fusion-containing strains streaked on LB agar plates was recorded with a ChemiDoc XRS system equipped with Quantity One 1-D analysis software (Syngene Chemi Genius), with an integration time of 30 s.

### Transposon Mutagenesis

Transposon mutagenesis was performed as described by Simms and Mobley (2008). Briefly, the MGN-617/pLOF-Km donor strain and recipient strain CFT073 carrying *fimS* phase variable reporter were cultured overnight (O/N) at 37°C in LB with appropriate antibiotics and supplements. Mixed cultures were prepared as a 1:4 donor-to-recipient ratio, placed onto LB agar plates supplemented with IPTG and DAP, and incubated O/N at 37°C. After incubation, cells were suspended in 1 ml of PBS, washed twice in PBS, serially diluted, and plated onto LB agar supplemented with kanamycin and incubated O/N at 37°C to select for the recovery of kanamycin-resistant transposon mutants of the CFT073 *fimS-lux* recipient strains. Colonies were screened for susceptibility to ampicillin to confirm loss of pLOF-Km.

### Measurement of Luminescence of Insertion Mutants in CFT073 Carrying *PfimA*-*lux*

Transposon mutants of CFT073 carrying *fimS-lux* were cultured at 250 rpm in 150 μL of LB in a 96-well plate (Corning White With Clear Flat Bottom), and luminescence was measured at O.D._600nm_. In total, 5,904 transformants were analyzed. The luminescence readings were normalized to the O.D._600_ values to account for any differences in growth. Mutants with disrupted genes that resulted in higher or lower levels of luminescence than the WT *pfim*-*lux*-fusion containing strain, were confirmed phenotypically by quantification of type 1 fimbriae by yeast agglutination assays as described below. Mutants of interest were then streaked on LB agar over three successive rounds of subculture and then stored individually in 25% glycerol at −80°C.

### Evaluation of Type 1 Fimbriae Production

The production of type 1 fimbriae was determined by yeast agglutination assay (Crépin et al., 2008). Briefly, the transposon mutants were cultured at 37°C in LB broth or human urine to mid-log phase. In our experiment, log-phase (period of steady-state growth in LB) is estimated to occur at OD_600_ (optical density at 600 nm) between 0.6 and 0.8 However, for growth in urine, cells reach a stationary phase at an OD_600_ of 0.5–0.9. As such, we used a growth of cells to an OD_600_ of 0.6 for cells for mid-log growth in LB and an OD_600_ of 0.4 for mid-log growth in human urine. Following centrifugation, 40 μl of an initial suspension of ~2 × 10^11^ cells ml^−1^ in PBS was transferred and serially diluted 2-fold in microtiter wells containing equal volumes of a 3% commercial yeast suspension in PBS. After 30 min of incubation on ice, yeast aggregation was monitored visually, and the agglutination titer was recorded as the most diluted bacterial sample giving a positive aggregation reaction.

### Site-Specific Integration of Tn*10*

Genomic DNA of 32 clones was extracted from cultures using phenol-chloroform. DNA was sequenced at the Génome Québec Innovation Centre, McGill University. DNA concentrations were determined using the Quant-iT™ PicoGreen^®^ dsDNA Assay Kit (Life Technologies). DNA samples were generated using the NEB Next Ultra II DNA Library Prep Kit for Illumina (New England BioLabs) as per the manufacture protocol. TruSeq adapters and PCR primers were purchased from IDT. Size selection of libraries containing the desired insert size was obtained using SPRI select beads (Beckman Coulter). Briefly, genomic DNA was fragmented and tagged with adapter sequence via one enzymatic reaction (tagmentation). We initially amplified by PCR the region between the end of the insertion (primer Tn_pLOF-Km-CS1:(5′ACACTGACGACATGGTTCTACAcgttgcgctgcccggattac 3′ [transposon-specific sequence is in lowercase])), and the Illumina adapter with primer 2 (5′ TACGGTAGCAGAGACTTGGTCTCTAGCATAGAGTGCGTAGCTCTGCT 3′) to enrich for transposon insertion sites and allow multiplex sequencing. The thermocycler program was 94°C for 2 min, 94°C for 30 s, 55°C for 30 s 72°C for 30 s for 33 cycles and 72°C for 7 min. Each library was prepared with a unique Illumina barcode. We amplified this region to add the Illumina adapters for MiSeq sequencing: PE1-CS1 (AATGATACGGCGACCACCGAGATCTACACTGACGACATGGTTCTACA) and primer 2. The libraries were then pooled in equimolar concentration and sequencing was performed on an Illumina MiSeq using the MiSeq Reagent Kit v2 Kit (500-cycles).

After determining the location of the transposon, the clones in the pool carrying the specific mutations were determined using a primer complementary to the transposon end and another primer complementary to the identified transposon-interrupted gene. Following DNA amplification of each clone by PCR, we were able to determine which specific clones contained some of the identified site-specific insertions.

### Construction of Site-Directed Mutants and Complementation of Strains

Mutations were introduced by lambda-red recombination as described using plasmids pKD3 and pKD4 as templates for chloramphenicol and kanamycin resistance cassettes, respectively (Datsenko and Wanner, 2000). Primers are listed in Supplemental Table 1. Antibiotic cassettes flanked by FLP recombination target (FRT) sites were excised by introduction of vector pCP20 expressing the FLP recombinase (Cherepanov and Wackernagel, 1995).

### Preparation of Fimbrial Extracts and Western Blotting

Preparation of fimbrial extracts and western blotting were performed as described previously (Crépin et al., 2008), with anti-type 1 fimbriae serum from *E. coli* strain B_AM_. Briefly, after the growth to log phase, the bacteria were harvested and resuspended in 5 ml of 150 mM NaCl-0.5 mM Tris-HCl (pH 7.8). Following incubation at 56°c for 1 h and centrifugation (3,000 × *g* for 10 min), the aliquot was precipitated with 10% trichloroacetic acid. Followed by centrifugation 20,000 × *g* for 15 min at 4°C, the pellet was washed twice with 0.5 M Tris-HCl-0.5 M EDTA (pH 12.0) and resuspended in 0.5 M Tris-EDTA.

Further, fimbrial extracts were separated by sodium dodecyl sulfate-polyacrylamide gel electrophoresis, the proteins were stained with Coomassie brilliant blue and transferred to a nitrocellulose membranes (Bio-Rad) for 60 min at 100 V. The membrane was blocked with supplemented with 0.05% Tween 20 (Pierce). Incubations with primary rabbit anti-*fim* (1:5,000) and secondary goat anti-rabbit (1:25,000) antibodies were carried out for 1 h at room temperature. SuperSignal West Pico chemiluminescent substrate (Pierce) was used for detection.

### Detection and Quantification of the On/Off State of the *fimS* Region

The orientation of the *fimS* region was determined as described previously (Müller et al., 2009). Briefly, the *fimS* region was PCR amplified with the primers CMD1258 and CMD1259 (see Supplemental Table 1), to produce a 650 bp fragment. The DNA was then digested with HinfI and analyzed on a 2% agarose gel. Following digestion, the ON orientation produces fragments of 128 and 522 bp, whereas the OFF orientation generates fragments of 411 and 239 bp. Quantification of the ratio of cells in ON or OFF position was performed as described (Wu and Outten, 2009). The WT strain was cultured statically for 48 h at 37°C for use as a control for increased orientation of the ON position. The WT strain was also cultured for 24 h on LB agar plates at room temperature as a control to favor orientation in the OFF position.

### Experimental UTI in CBA/J Mice

The animal study was reviewed and approved by the animal ethics evaluation committee—Comité Institutionel de Protection des Animaux (CIPA No 1608-02) of INRS. The murine experimental UTIs were carried out as described previously (Hagberg et al., 1983), using a single-strain infection model. Prior to inoculation, strains were grown for 16 h at 37°C with shaking (250 rpm) in 55 ml of LB medium. Six-weeks-old CBA/J female mice were inoculated through a catheter inserted in the urethra with 20 μl of the pellet containing 2 × 10^9^ CFU of either UPEC strain CFT073, CFT073 Δ*yqhG* (QT5178) or the complemented strain (QT5235). After 48 h, mice were euthanized; kidneys and bladders were sampled, homogenized, diluted, and plated on MacConkey agar for enumeration of colonies.

### Adhesion Assays

5,637 human bladder cells (ATCC HTB-9) were grown in RPMI 1640 medium (Wisent Bioproducts) supplemented with 10% fetal bovine serum, 2 mM l-glutamine, 10 mM HEPES, 1 mM sodium pyruvate, 4.5 g/liter glucose, and 1.5 g/liter sodium bicarbonate. For the assays 5,637 cells were grown to confluency in RPMI 1640 and 2 × 10^5^ cells/well were distributed in 24-well plates. Strain CFT073 and isogenic mutants were grown in LB medium at 37°C to the mid-log phase of growth (O.D. 0.6). Immediately before infection, cultures were washed with PBS, suspended in medium and inoculated at a multiplicity of infection (MOI) of 10 CFU per epithelial cell. Bacteria-epithelial cell contact was enhanced by a centrifugation at 600 × g for 5 min. After 2 h, cells were washed three times and lysed with PBS−0.1% sodium deoxycholate (DOC), serially diluted, and plated on LB agar plates. Quantification of cell-associated bacteria was performed as previously described (Martinez et al., 2000). To block adherence mediated by type 1 fimbriae, 2.5% α-d-mannopyranose was added.

### Motility Assay

Motility assays were as previously described (Lane et al., 2005) with modification. Following overnight growth at 37°C, strains were cultured at 37°C in LB broth to mid-log phase. Strains were stabbed into the surface of soft agar (1% tryptone, 0.5% NaCl, 0.25% agar) using an inoculating needle. Care was taken not to touch the bottom of the plate during inoculation to ensure only swimming motility was assessed. After 16 h of incubation, the diameters of motility zones were measured. Three independent motility experiments for each strain were performed. Results were analyzed using a paired *t*-test.

### Growth Under Conditions of Osmotic Stress

Strains were tested for growth under conditions of osmotic stress using NaCl or urea. Cultures were diluted 1:100 from overnight cultures grown in LB, grown until mid-log phase with shaking. They were serially diluted and plated on LB agar alone and LB agar supplemented with 0.3 M NaCl, 0.6 M NaCl, 0.3 M urea, or 0.6 M urea. Colonies were enumerated, and growth under each condition was compared to growth on LB agar.

### Hydrogen Peroxide Sensitivity Assay

Sensitivity to H_2_O_2_ was determined by using an agar overlay diffusion method (Boyer et al., 2002). Briefly, overnight cultures were used to inoculate (1/100) fresh LB medium, and incubated until the O.D._600_ reached 0.6. Then, 100 μl of each culture were mixed with 3 ml molten top agar and poured onto an LB agar plate. A 6-mm-diameter Whatman filter disk containing 10 μl 30% H_2_O_2_ was placed on the agar surface and plates were incubated overnight at 37°C. Inhibition zone diameters were then measured.

### Statistical Analyses

Statistical tests were obtained using the Prism 7.04 software package (GraphPad Software). Statistically significance between two groups was determined by unpaired *t*-test and comparison among three or more groups was obtained by one-way analysis of variance (ANOVA). For the independent infections, comparisons of the CFU mL^−1^ or CFU g^−1^ distributions were analyzed using the Mann–Whitney test.

## Results

### The Single-Copy Integrated CFT073 *fimS-lux* Reporter System

**Figure 1:**
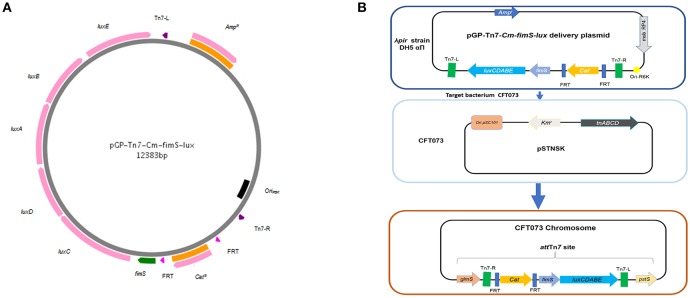
Methods for site-specific insertion of *fimS-lux* fusions using mini-Tn 7-*lux* vector. **(A)** The mobilizable suicide vector pGP-Tn*7*-*Cm-fimS-lux* contains the conjugative transfer Mob RP4 and the *ori* R6K. A multiple cloning site is integrated between the two Tn*7* ends, where the promoterless *lux* reporter system is fused to the fimbrial promoter region, *fimS*. **(B)** Tn7-based transposition at the chromosomal *att*Tn7 site was achieved by conjugation, where the donor strain (*E. coli* SM10λ*pir*) harbored the mini-Tn7 vector and the recipient strain CFT073 contained the thermosensitive suicide vector pSTNSK pSC101-temperature sensitive origin and transposases *tnsABCD*. The Tn*7* transposon integrates at the site-specific *att*Tn*7*, located downstream of the highly conserved *glmS* gene.

To identify systems that alter the expression of type 1 fimbriae, we used a promoterless *lux* reporter system fused to the *fim* type 1 fimbriae promoter region, *fimS*. A *luxCDABE* reporter system originally from *Photorhabdus luminescens* (Allen, 2000) was used. The system encodes all the enzymes needed to produce a luminescent signal. The *lux* genes were introduced into the pGP-Tn7-*Cm* vector. An optimized ribosome binding site, *RBS*, was added to plasmid pIJ461 to generate pIJ514 vector. Further, we generated phase variable, *pfimA phase variable-lux* (pIJ518) to measure the expression of type 1 fimbriae in various conditions (Figure 1A). Using the same approach as described by Gunther et al. (2002), point mutations were introduced in order to lock the promoter in the ON- and OFF-position, respectively to generate *pfimA L-ON-lux* (pIJ516) and *pfimA L-OFF-lux (*pIJ517) (Figure 1).

### Analysis of the *fim-lux* Fusions in UPEC CFT073 Grown Under Different pH Conditions

**Figure 2:**
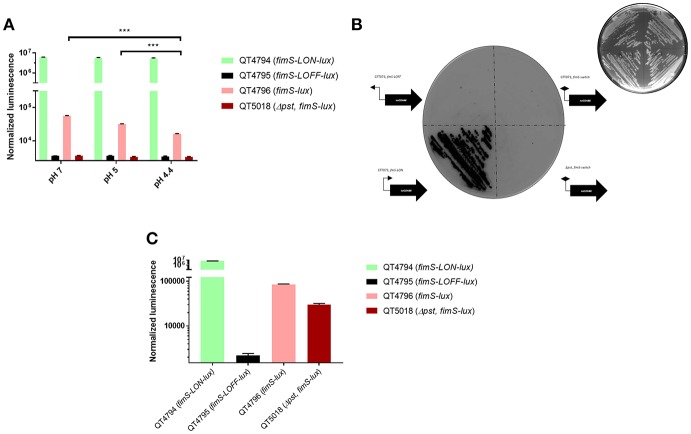
Response of the *lux* reporter system in exponential-phase, overnight cultures and on agar. **(A)** Effects of pH on *fimS-*phase variable, *fimS-*Locked ON and *fimS-*Locked OFF expression was determined with *luxABCDE* transcriptional fusions in strain CFT073. Luminescence was normalized to the O.D._600nm_ of the culture compared to CFT073; means ± standard deviations are indicated from at least three separate runs. **(B)** Inverted darkfield image of luminescence emitted on plates by CFT073 and *pst* reporter strains carrying *PfimA* from the constitutively expressed promoter (*fimS*-*LON*) or inverted promoter (*fimS-LOFF*) or switch variable (*fimS*-phase variable) direction with respect to *luxCDABE*. Colonies were grown overnight at 37°C and imaged using white light 400 ms (top right corner) or luminescence imaging for 30 s exposure time. Exposure images were acquired through ChemiDoc XRS system using high sensitivity chemiluminescence settings. **(C)** Response of the *lux* reporter system in overnight cultures in LB broth. Statistical significance was calculated by the Student *t-*test: ^*^P < 0.05; ^**^P < 0.005; ^***^P < 0.0001.

To better characterize the reporter system upon dynamic transcriptional changes, we set out to assay the response kinetics of the *pfimA* promoter, driving the expression of the type 1 fimbriae using the *lux* reporter system. This promoter has been extensively studied in *E. coli* and used to validate a *lux* reporter system and *lacZYA* fusion implemented on a single copy number plasmid (Schwan et al., 2002; Schwan and Ding, 2017). Thus, it has been reported that growth conditions play a substantial role in the ability of *E. coli* cells to undergo phase variation and alter expression of type 1 fimbriae. Of note, transcription of all of the *fim* genes was shown to be repressed in a low pH environment (Schwan et al., 2002). Following a similar protocol, the *fim-lux* reporter containing variant of CFT073 or its isogenic *pst* mutant was grown in LB adjusted at different pH conditions ranging from 4.4 to 7. To verify any differences in expression, the *E. coli* cells were grown to mid-logarithmic phase (OD_600_ of 0.6). The *fimS-*Locked ON fusion had the highest expression level and did not vary regardless of the pH during growth (Figure 2A). However, the neutral pH influenced the expression of the *fim* switch. A shift from pH 4.4 to a neutral pH of 7 in LB media resulted in a 2-fold increase in expression of the *fimS* promoter. Decrease in pH also diminished the production of type 1 fimbriae (Supplemental Figure 1). In addition, no significant change in growth rates was observed for growth of the CFT073 parent strain and the P*fimA-*Locked ON and P*fimA* variable *lux* derivatives, suggesting that the expression of the *lux* operon and its gene products had no adverse effects on bacterial growth (Supplemental Figure 2).

CFT073 derivatives carrying the *PfimA* promoter in either direct (*PfimA-*Locked ON) or opposite (*PfimA-*Locked OFF) orientation with respect to the *luxCDABE* operon were streaked on LB agar plates, together with the CFT073 strain and a *pst* mutant carrying the *PfimA* phase-variable *lux* fusion. Stronger emission was detected from the *fimS-*Locked ON fusion (Figure 2B), with single colonies producing a strong signal. By contrast, there was no luminescence signal detected in the negative control carrying the *PfimA-Locked-OFF* fusion or the CFT073 strain or its *pst* mutant carrying the *PfimA*-phase-variable-*lux fusion*, although a dim light emission could be detected for CFT073 containing the *PfimA*-phase-variable fusion after longer exposure. In addition, the stability of the *fimS-*Locked-ON*-lux* strain was evaluated after 10 passages without antibiotic selection and the maintenance and luminescence expression of this fusion was found to be stable.

The expression of *lux* derivatives was also compared in LB liquid media overnight. As expected, there was a high signal for the *fimS-*Locked ON strain (QT4794), no signal for the *fimS*-Locked *OFF* strain (QT4795) and an intermediate signal for the *fimS-lux* variable strain (QT4796) (Figure 2C). In contrast, the signal for Δ*pst, fimS-phase variable-lux* (QT5018) increased significantly (Figure 2C) when compared with the same mutant at OD_600_ 0.6 (Figure 2A). This result was similar to what was previously observed (Supplemental Figure 1) (Crépin et al., 2012b).

### Screening for Transposon Mutants With Increased or Decreased *lux* Expression and Altered Production of Type 1 Fimbriae

**Figure 3:**
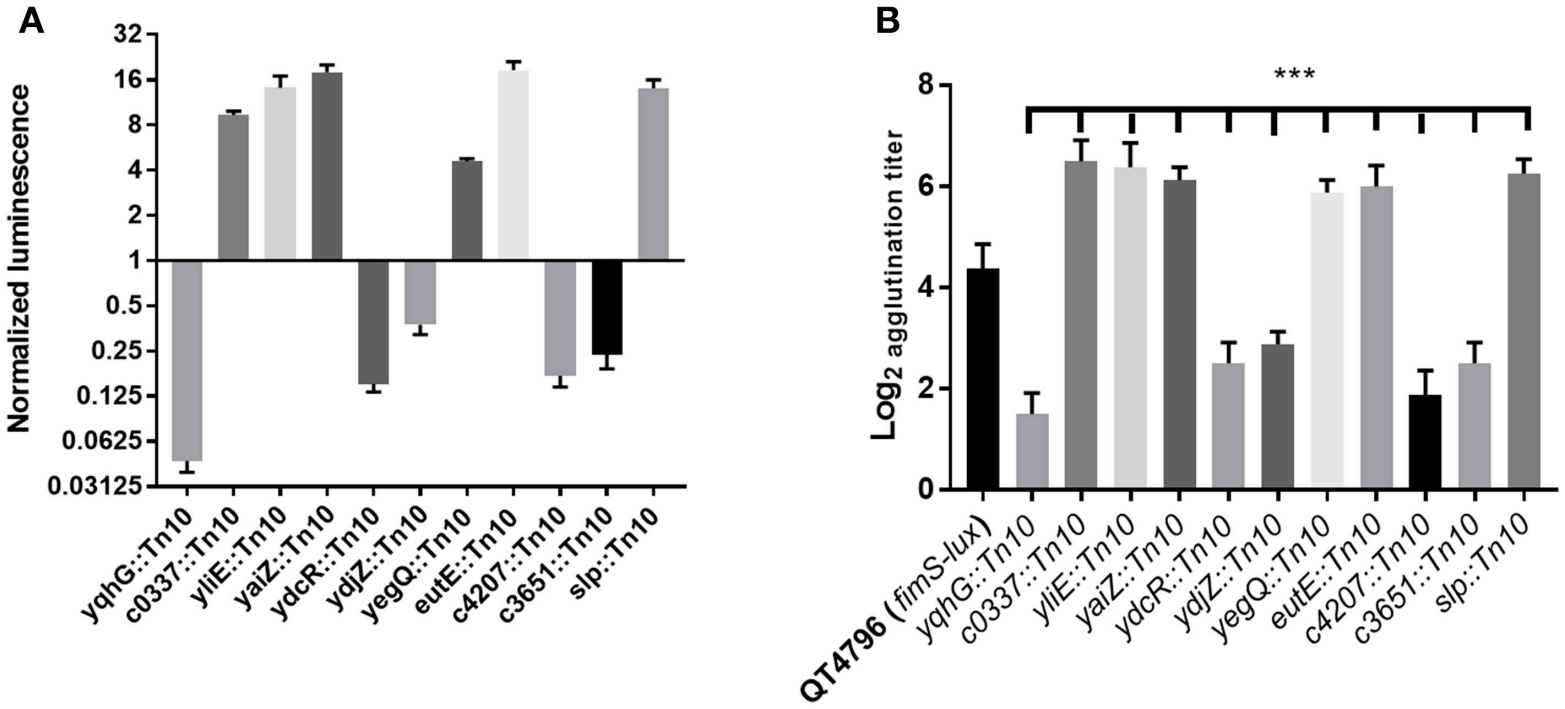
Screening of transposon mutants based on *lux* expression and the production of type 1 fimbriae. **(A)** A decrease or increase of luminescence corresponding to the expression of type 1 fimbriae was observed in the transposon mutants, **(B)** Production of type 1 fimbriae in transposon mutants cultured to the mid-log phase in LB broth determined by yeast agglutination. The QT4796 (*fimS-lux*) strain was used as a control and showed baseline luminescence. Results are the mean values and standard deviations for three biological experiments. Statistical significance was calculated by the Student *t*-test: ^*^P < 0.05; ^**^P < 0.005; ^***^P < 0.0001.

To identify genes affecting the expression of type 1 fimbriae, *E. coli* CFT073 carrying the *fimS* phase-variable reporter (Figure 1B) was subjected to transposon mutagenesis. A mini-Tn*10* transposon carrying a kanamycin resistance marker pLOF/Km (Herrero et al., 1990) was randomly inserted into the bacterial chromosome. A transposon bank was screened for luminescence. Clones demonstrating increased or decreased light emission were identified. Transposon insertions that produced high or low levels of luminescence may identify genes encoding transcriptional repressors or activators of *fim*. Transposon insertions that result in low levels of luminescence suggest that the disrupted genes may promote *fim* transcription and production of type 1 fimbriae. A total of 5,904 transposon mutants were generated and stored individually in 96-well plates stored at −80°C. The mutants were assessed for luminescence at O.D._600nm_ 0.6 with shaking at 37°C (Figure 3A). A subset of 65 transposon mutants demonstrating the highest and lowest levels of luminescence compared to the control CFT073 (attTn7 *PfimA-lux*) were further evaluated. Mutants that produced less luminescence than the control strain were further tested for levels of production of type 1 fimbriae by yeast agglutination assay (Crépin et al., 2012b). From this secondary screen, 48 transposon mutants were confirmed and there was at least a 4-fold increase or decrease in yeast agglutination (p <0.0001) (Figure 3B). Some of these mutants are currently being investigated to determine the transposon insertion site locations. In addition, 32 of the individual mutants were pooled and their transposon insertions were determined (Supplemental Figure 3) as described in the Materials and Methods section.

### Identification of Mutations Affecting Type 1 Fimbriae Expression

Analysis of the 32 mutants generated 6,733 sequence reads that mapped to the CFT073 genome. The insertions were linked to genes involved in protein fate and synthesis, energy metabolism, adherence, transcriptional regulation, and transport, regulatory functions, etc. (Table 2). A number of transposon mutations were also inserted in genes predicted to encode proteins of unknown function (Supplemental Figure 3).

**Table 2:** Transposon mutants with altered p*fimA::lux* expression in CFT073^*^.

**Insertion site^a^**	**CDS**	**Gene symbol**	**Gene product description**
**ENERGY METABOLISM**			
*yecK*	*c2287*	*yecK*	Cytochrome c-type protein torY
*eutE*	*c2980*	*eutE*	Ethanol amine utilization protein eutE
*sseB*	*c3047*	*sseB*	Serine sensitivity enhancing B (SseB)
*nadB*	*c3098*	*nadB*	L-aspartate oxidase
*iucC*	*c3625*	*iucC*	IucC protein
*tdcB*	*c3875*	*tdcB*	Threonine dehydratase
**FIMBRIAL ADHESINS, TRANSPORTERS, OR OUTER MEMBRANE PROTEINS**			
*ydeS*	*c1933*		ydeS—fimbrial-like protein ydeS precursor (minor subunit proteins F9 fimbriae)
*aufG*	*c4207*	*aufG*	Putative fimbrial adhesin precursor
*fimD*	*c5396*	*fimD*	Outer membrane usher protein fimD precursor
*dnaJ*	*c0020*	*dnaJ*	Chaperone protein DnaJ
*yaiO*	*c0467*		Outer membrane protein YaiO
*ydcS*	*c1864*	*ydcS*	ABC transporter periplasmic-binding protein (polyhydroxybutyrate synthase)
*nmpC*	*c2348*	*nmpC*	Outer membrane porin protein nmpC precursor
*emrK*	*c2904*	*emrK*	Multidrug resistance protein K
Adjacent to *kpsM*	*-c3698*	*kpsM*	Capsule synthesis
*slp*	*c4304*	*slp*	Outer membrane protein slp precursor
*ytfR*	*c5326*	*ytfR*	Putative ATP-binding component of a transport system
**REGULATORS**			
*yliE*	*c0918*	*yliE*	Putative c-di-GMP phosphodiesterase PdeI
*lrp*	*c1026*	*lrp*	Leucine-responsive transcriptional regulator
*rstA*	*c2000*	*rstA*	DNA-binding transcriptional regulator RstA
	*c3750*		Putative regulator
*kguS*	*c5041*	*KguS*	α-ketoglutarate utilization sensor
*fimB*	*c5391*	*fimB*	FimB recombinase regulator for *fimA*
**UNKNOWN FUNCTION**			
	*c0337*		Putative conserved protein
*yaiZ*	*c0486*	*yaiZ*	Hypothetical protein
	*c1269*		Hypothetical protein
	*c1555*		Putative DNA N-6-adenine-methyltransferase of bacteriophage
*ynjA*	*c2154*	*ynjA*	Hypothetical protein
*yegQ*	*c2611*	*yegQ*	Putative protease yegQ
*yqhG*	*c3747*	*yqhG*	Hypothetical protein YqhG precursor
Adjacent to *yqhG*	*c3746-c3747*	*yqhG*	Hypothetical protein YqhG precursor

* *List includes insertions identified that had at least 4-fold greater or 4-fold less relative light units compared to the CFT073 control level of lux expression. Genetic locus with the closest match to the sequence interrupted by the transposon in each mutant. Locations of specific Tn insertions are presented in Supplemental Figure 3*.

a *Genes in bold are present in the genome of E. coli K-12 strain MG1655 genome*.

### Insertions Within Genes Contributing to Amino Acid Biosynthesis and Metabolism

Several clones carried an insertion within genes with metabolic functions. For instance, one of these clones was inserted in *tdcB* (Table 2) that encodes a catabolic threonine dehydratase involved in the first step in threonine degradation. It is one of several enzymes carrying out the first step in the anaerobic breakdown of L-threonine to propionate (Umbarger and Brown, 1957). However, TdcB is activated by cAMP. Following a transition from aerobic to anaerobic growth, cAMP levels rise dramatically, which leads to increased expression of *tdcB* and consequently high levels of catabolic threonine deaminase (Hobert and Datta, 1983).

Similarly, another insertion was in *nadB* (Tritz et al., 1970) which is involved in NAD biosynthesis under both aerobic and anaerobic conditions (Messner and Imlay, 2002). Another mutant had an insertion in a pathogen-specific iron acquisition gene, *iucC*, which encodes a protein required for synthesis of aerobactin (de Lorenzo and Neilands, 1986). Likewise other insertion sites were *yecK*, encoding a membrane anchored pentaheme *c*-type cytochrome involved in an anaerobic respiratory system (Gon et al., 2000); *eutE* which codes for a protein that increases the level of acetylating acetaldehyde dehydrogenase activity (Rodriguez and Atsumi, 2012) and *sseB* involved in increased rhodanese activity (Hama et al., 1994).

### Insertions Within Genes Encoding Other Fimbrial Adhesins, Transporters, or Outer Membrane Proteins in *E. coli* CFT073

A number of mutants with altered *lux* expression were found to contain insertions in genes encoding fimbriae, outer membrane proteins and transport proteins. Among these mutants, three carried insertions in fimbrial systems. One insertion was identified in *fimD* which is the usher of the chaperone-usher pathway of type 1 fimbriae (Supplemental Figure 3) (Klemm and Christiansen, 1990), the other mutant had an insertion in *aufG* which codes for a putative fimbrial-like adhesin protein, and another insertion was identified in c1933 (*ydeS*) encoding the minor fimbrial subunit of F9 fimbriae (Table 2). These loci have been previously characterized (Buckles et al., 2004; Ulett et al., 2007). We also identified insertions in genes encoding a putative ABC transporter periplasmic binding protein, *ydcS*, a predicted outer membrane protein, *yaiO*, and an outer membrane porin, *nmpC* (Table 2). Chaperone genes involved in protein fate, such as *dnaJ* coding a chaperone protein were also identified. DnaJ acts as a sensor for non-native proteins (Siegenthaler and Christen, 2006) and is involved in many cytoplasmic events, such as promotion of protein folding and translocation of nascent polypeptides (Hartl, 1996).

### Insertions Within Regulatory Genes

Further, sequencing also identified insertions in well-known regulatory genes, such as *lrp* (Calvo and Matthews, 1994). Insertion within *fimB*, which mediates switching in both directions was also identified. An insertion was identified within the *c0918* (*yliE*) gene (Table 2). *yliE* encodes a hypothetical conserved inner membrane protein which contains a phosphodiesterase EAL domain. This protein is involved in hydrolysis of the bacterial second messenger cyclic di-GMP (c-di-GMP), a key factor in processes, such as flagellar motility, biofilm formation, the cell cycle, and virulence of pathogenic bacteria (Jenal and Malone, 2006; Römling et al., 2013). Similarly, another clone contained an insertion in *c5041* (*kguS*) which was identified as a sensor protein of a two-component signaling system involved in α-ketoglutarate utilization. This system is involved in utilization of α-ketoglutarate, an abundant metabolite during UPEC infection, which regulates target genes that encode α-ketoglutarate dehydrogenase and a succinyl-CoA synthetase. This two-component signaling system has been shown to be important for UPEC fitness during UTI (Cai et al., 2013). Also, *rstA*, a member of the two-component regulatory system RstB/RstA (Calvo and Matthews, 1994) was disrupted in one of the mutants.

### Insertions Within Hypothetical Genes

**Figure 4:**
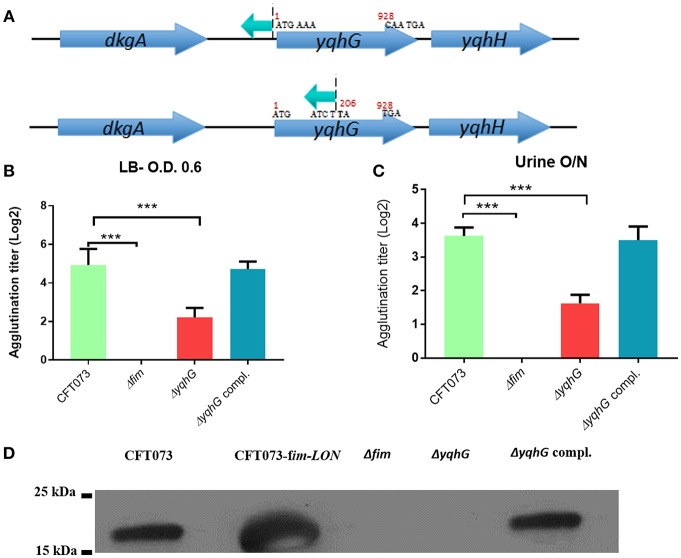
Inactivation of the *yqhG* gene reduced expression of type 1 fimbriae. **(A)** Schematic representation of a transposon insertion which caused a decrease in expression of type 1 fimbriae. **(B)** Production of type 1 fimbriae in strains cultured to the mid-log phase of growth in LB broth and O/N in urine. The Δ*fim* strain was used as a negative control and showed no agglutination. **(C)** Production of type 1 fimbriae in strains cultured O/N in human urine. **(D)** Western blot of fimbrial extracts of strains cultured to the mid-log phase of growth in LB broth. Results are the mean values and standard deviations for six biological experiments. Statistical significance was calculated by the Student *t*-test: ^*^P < 0.05; ^**^P < 0.005; ^***^P < 0.0001.

Several of the mutations disrupted genes predicted to encode hypothetical proteins of unknown function, and the roles of such genes for *E. coli* physiology as well as their influence on expression of type 1 fimbriae and UPEC pathogenesis are unknown (Table 2). Sequence analysis identified an independent insertion within the *yqhG* gene and an insertion immediately upstream of the *yqhG* coding region (Figure 4A), strongly suggesting the involvement of YqhG in type 1 fimbriae production. Thus, we focused the rest of our investigation on this gene of unknown function and its role for production of type 1 fimbriae and UPEC pathogenesis.

### Disruption of *yqhG* Reduces Expression of Type 1 Fimbriae

A transposon was inserted in the opposite orientation of *yqhG* in one mutant and in the middle of the gene in another mutant (Figure 4A), suggesting that the *yqhG* gene was involved in regulation of expression of type 1 fimbriae. Since type 1 fimbriae contribute to UPEC pathogenicity (Nielubowicz and Mobley, 2010), the production of type 1 fimbriae was then evaluated by yeast agglutination at the mid-log phase of growth in LB (O.D 0.6) and urine (O.D 0.4). As expected, mutations within *yqhG* had an effect on the production of type 1 fimbriae (Figure 4B). After mid-log growth with shaking in LB, and overnight growth in human urine with shaking, the agglutination titer of CFT073Δ*yqhG* was reduced by 4-fold as compared to strain CFT073. Agglutination titers of the complemented mutant regained titers similar to that of the WT strain (Figure 4B). To confirm that yeast agglutination was mediated by type 1 fimbriae, the assay was also performed in the presence of 2.5% mannopyranose, which blocks type 1 fimbriae-mediated agglutination. As expected, no yeast agglutination was observed when 2.5% mannopyranose was added to the bacteria. These results indicate that the mutation of *yqhG* caused a substantial decrease of type 1 fimbriae expression. Since the *yqhG* mutant demonstrated a defect in type 1 fimbriae production (Figure 4B), we further investigated the ability of the mutant to grow in human urine. Loss of the *yqhG* in strain CFT073 did not affect growth in human urine.

To confirm that production of type 1 fimbriae was reduced in the *yqhG* mutant, Western blotting against the type 1 major subunit FimA was determined. Western blotting confirmed an important decrease of the FimA protein in the *yqhG* mutant compared to parent strain CFT073 and the complemented strain (Figure 4D).

### The *yqhG* Mutant Demonstrates Reduced Adherence to Human Bladder Epithelial Cells

**Figure 5:**
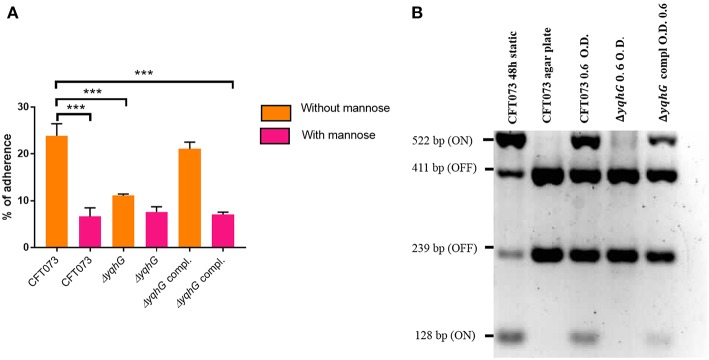
**(A)** Effect of inactivation of *yqhG* and production of type 1 fimbriae on adherence of uropathogenic *E. coli* CFT073 to human bladder epithelial cells *in vitro*. Adherence of strain CFT073 and its derivatives to human 5,637 bladder epithelial cells in the presence or absence of 2.5% α-d-mannopyranose was determined. **(B)** Effect of inactivation of *yqhG* on orientation of the *fim* promoter switch (*fimS*) *in vitro*. The *fimS* region was PCR amplified, and the product was digested with HinfI. Fragments of different sizes indicate the ON or OFF orientation. All results shown are the mean values and standard deviations for four biological experiments. Statistical significance was calculated by the Student *t*-test **(A)**. ^*^P < 0.05; ^**^P < 0.005; ^***^P < 0.0001.

The adherence of the *yqhG* mutant to 5,637 human bladder epithelial cells was compared to that of the parental strain CFT073. Figure 5A shows that the adherence of the Δ*yqhG* mutant was reduced ~2-fold compared to that of WT strain. Further, the decrease in epithelial cell adherence was rescued by complementation of the *yqhG* gene. Addition of 2.5% α-d-mannopyranose to block the effect of type 1 fimbriae greatly reduced cell association of all strains tested in the cell association assay (Figure 5A).

To determine if the reduction of type 1 fimbriae was due to orientation bias of the phase-variable promoter, we determined orientation of the *fimS* promoter region. The orientation of *fimS* was evaluated in strains grown under agitation to mid-log phase in LB broth. Using the procedure described by Stentebjerg-Olesen et al. (2000), we observed that the *fim* promoter clearly had an increased bias for the OFF position in the *yqhG* mutant (Figure 5B).

### The *yqhG* Mutant Demonstrates Reduced Bladder and Kidney Colonization in Mice

**Figure 6:**
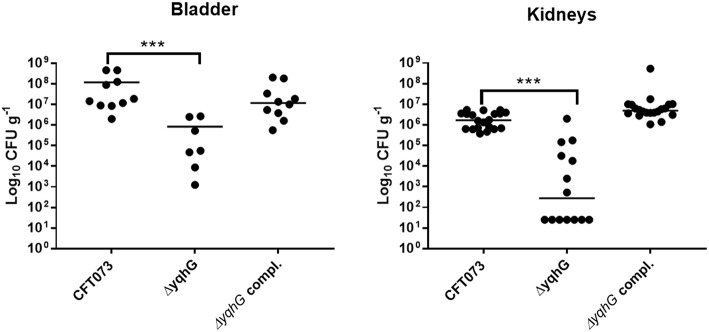
Deletion of *yqhG* reduces colonization of the murine urinary tract. CBA/J mice were infected transurethrally and the animals were euthanized, and organs were collected 48 h post-infection. Each data point represents a sample from an individual mouse, and horizontal bars indicate the medians. Two independent infections were performed: with CFT073 WT and CFT073Δ*yqhG* and with CFT073 WT and CFT073Δ*yqhG-*Tn7T-Cm::*yqhGH*. Each kidney was sampled separately (Mann-Whitney test). ^*^P < 0.05; ^**^P < 0.005; ^***^P < 0.0001.

Type 1 fimbriae are important for ExPEC colonization of the bladder during UTIs. Since the *yqhG* mutant demonstrated decreased type 1 fimbriae production, we tested its capacity to cause urinary tract infection in the CBA/J mouse model. Forty-eight hours after urethral inoculation, the *yqhG* mutant was attenuated 100-fold in bladder and 10,000-fold in kidneys (*P* < 0.0001) compared to the WT parent strain (Figure 6). Further, the complemented mutant regained the capacity of colonization. This reduction in colonization from inactivation of *yghG* could be due to reduced production of type 1 fimbriae, and potentially other changes in the *ΔyqhG* mutant that could decrease colonization of the murine urinary tract (Figure 6).

### The *yqhG* Mutant Demonstrates Increased Motility

**Figure 7:**
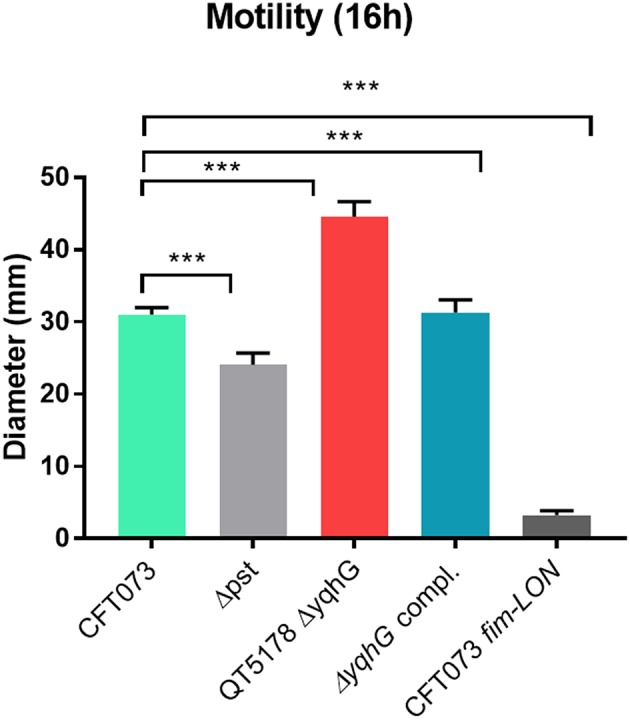
Effect of deletion of *yqhG* on motility. Diameters of swimming motility of CFT073, mutants and complemented mutant. Data represent the averages of five separate experiments. Error bars represent the SEM. Significant differences in motility between mutants and complemented mutants were determined using a paired Student *t*-test. ^*^P < 0.05; ^**^P < 0.005; ^***^P < 0.0001.

Since flagella and swimming motility play a pivotal role in colonization and persistence in the urinary tract (Lane et al., 2005), we investigated whether the loss of *yqhG* affected swimming motility. The *yqhG* mutant was considerably more motile than the UPEC CFT073 parental strain. Complementation of the *yqhG* mutant (strain QT5235) effectively restored motility to wild-type levels. Further, a *fimS* Locked-ON reference strain was shown to be non-motile in the swimming assays (Figure 7).

### *yqhG* Contributes to Oxidative Stress Resistance

During the course of a UTI, UPEC come across a variety of environmental stresses that can potentially limit survival and growth (Mulvey et al., 2000). Envelope stress response pathways are likely to be critical for UPEC, in order to detect and respond to potentially harmful environmental insults during the course of infection. In an effort to determine the mechanism by which *yqhG* influences type 1 fimbriae expression, we first sought to determine if this gene is involved in hydrogen peroxide resistance. Our observations revealed that the Δ*yqhG* strain was more sensitive to oxidative killing compared to wild-type strain CFT073. This result indicates that *yqhG* contributes to resistance to oxidative stress mediated by hydrogen peroxide (Table 3). Further, screening of specific transposon mutants that were also shown to have decreased expression of type 1 fimbriae including clones with insertions in the *slp or yegQ* genes also showed increased sensitivity to hydrogen peroxide-mediated oxidative stress (Table 3).

**Table 3:** Growth inhibition zones of UPEC CFT073, isogenic mutants, and complemented strains exposed to 10 μl of hydrogen peroxide.

**Strain**	**Mean diameter inhibition zone (mm) in LB ± SD^a^**
CFT073	25.56 ± 0.49
Δ*pst*	**31.25** **±** **0.70**
Δ*pst* compl.	26.0625 ± 0.41
Δ*yqhG*	**28.62** **±** **0.51**
Δ*yqhG* compl.	25.12 ± 0.58
QT1324 (*oxyR::Km*)	**39.35** **±** **1.04**
QT4937 (*slp::Tn10*)	**28.93** **±** **0.67**
QT4940 (*yegQ::Tn10*)	**28.87** **±** **0.23**

*Data presented are the means ± standard deviations for eight independent experiments. The compound used was 10 μl of H_2_O_2_ (30%) on LB or agar plates*.

a *Mean of eight determinations per strain. All strains were tested in parallel each day. Values indicated in bold text are significantly different, P < 0.05, from the mean for the wild-type strain as calculated by Student's t-test*.

### The *pst* Mutant of UPEC CFT073 Is Also Sensitive to Osmotic and Oxidative Stress

**Figure 8:**
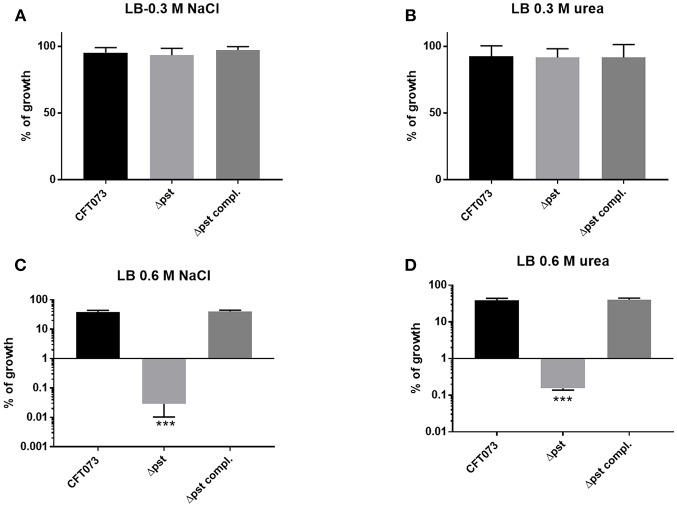
Growth in conditions of osmotic stress. Strains were grown under shaking in LB medium until mid-log phase (O.D._600_ 0.6) and plated on LB agar (taken as 100% growth) and **(A)** LB agar with 0.3M of NaCl, **(B)** 0.3M of urea, **(C)** 0.6M NaCl, and **(D)** 0.6M of urea. Graphs show the mean of growth relative to regular LB with standard error bars. Assays were performed three times in duplicates (Kruskal-Wallis test). ^*^P < 0.05; ^**^P < 0.005; ^***^P < 0.0001.

In a previous study, in avian pathogenic *E. coli* (APEC) strain χ7122, inactivation of the *pst* system mimicked phosphate-limiting conditions and caused pleiotropic effects (Lamarche et al., 2008). In the APEC *pst* mutant, membrane homeostasis was altered and included modification of phospholipids (Lamarche et al., 2008). Accordingly, the same mutant appeared to modulate the expression of some genes regulating antioxidant activities (Crépin et al., 2008). In the UPEC strain CFT073 *pst* mutant, attenuation of urinary tract virulence was shown to mainly be attributed to reduced production of type 1 fimbriae (Crépin et al., 2012b). As stress resistance is crucial for the survival of UPEC strains in the host, we also evaluated the capacity of the UPEC CFT073-derived *pst* mutant to resist oxidative and osmotic stresses. Sensitivity to hydrogen peroxide was analyzed from exponential growth cultures of CFT073 and the Δ*pst* derivative by using the H_2_O_2_ agar overlay diffusion method. Under phosphate-sufficient conditions (LB), the *pst* mutant was more sensitive to oxidative stress, as diameters of inhibition zones were significantly larger with the *pst* mutant than with the CFT073 parental strain (*P* < 0.001) (Table 2). Complementation of the Δ*pstCAB* mutant restored the wild-type phenotype (Table 3). Given the significant decrease in the colonization of the mouse bladder by the *pst* mutant, we also tested whether this strain displayed increased susceptibility to osmotic stress by using an established protocol (Pavanelo et al., 2018). We, therefore, tested the growth of the wild-type, *pst* mutant, and a complemented mutant on modified LB agar containing different concentrations of NaCl or urea. Both strains were able to grow on LB agar with 0.3 M NaCl (Figure 8A) and with 0.3 M urea (Figure 8B), and no strain could grow on LB agar with 1 M NaCl and 1 M urea. In contrast, the *pst* mutant was significantly more sensitive to 0.6 M NaCl (Figure 8C) and 0.6 M urea (Figure 8D) compared to parent strain CFT073. The complemented mutant grew similarly to the WT strain (Figure 8). Taken together, the *pst* mutant was found to show increased sensitivity to both oxidative and osmotic stresses.

## Discussion

The ability to adhere to host epithelial cells is an important factor for initial colonization and persistence during a UTI. Therefore, type 1 fimbriae are crucial for the establishment of UPEC infections (Schaeffer et al., 1987). Bacterial adherence to the uroepithelium limits the effect of shear forces produced by urine flow and thereby improves colonization (Thomas et al., 2002). Urine is considered to be a nutrient limiting environment with relatively low levels of available sugars and metabolites. Consequently, UPEC metabolism is tightly regulated and highly responsive to nutrient availability, and UPEC adapted to utilization of a wide range of nutrients from this nutrient-limited environment (Mann et al., 2017). Further, most bacteria are unable to thrive within the urinary tract environment due to its high osmolarity, elevated urea concentrations, low pH, and limited iron availability (Mann et al., 2017). Due to this transition, bacteria that enter the urinary tract encounter a harsh environment and are subject to numerous stresses, and stringent competition due to a drastic reduction in the abundance of nutrients. Further, environmental cues, such as pH and osmolality, have been shown to regulate *fim* genes and to affect the orientation of the *fim* switch (Schwan et al., 2002).

The expression of type 1 fimbriae is controlled by an invertible DNA element that upon inversion changes the promoter orientation through two site-specific recombinases, FimB and FimE. Thus, affecting the transcription of the *fimAICDFGH* genes (Abraham et al., 1985). This inversion phenomenon known as phase variation, reversibly switches between the expression of type 1 fimbriae (Phase-ON) and loss of expression (Phase-OFF) (Klemm, 1986). Besides the *fim* gene cluster, other genes and their gene products contribute to the expression of type 1 fimbriae. The global regulator histone-like nucleoid-structuring protein (H-NS), integration host factor (IHF), leucine-responsive protein (Lrp), and cyclic adenosine monophosphate (cAMP) receptor protein (CRP)/cAMP, control the expression of type 1 fimbriae directly and indirectly (Blomfield et al., 1997; Olsen et al., 1998; Kelly et al., 2006). Other proteins affect type 1 fimbriae expression in *E. coli* such us OmpX (Otto and Hermansson, 2004), IbeA, and IbeT (Cortes et al., 2008), although mechanisms of control are unknown. The regulatory alarmone, ppGpp, has been connected to the regulation of the *fim* operon (Åberg et al., 2006). Alteration of phosphate metabolism through inactivation of the phosphate specific transporter (*pst)* was shown to contribute to expression of type 1 fimbriae and attenuated UPEC virulence (Crépin et al., 2012b). Further, inactivation of *pst* was linked to increased production of the signaling molecule c-di-GMP, which in turn decreased the expression of type 1 fimbriae in UPEC CFT073 (Crépin et al., 2017). It has been shown that under a slightly acidic pH and low salt growth conditions found on the vaginal surface, that proteins, such as SlyA or RcsB may activate *fimB* and prevent H-NS from binding, allowing type 1 fimbriae to be expressed on the surface of the UPEC cells (Schwan et al., 2007; McVicker et al., 2011). Recently, the *treA* gene coding for a periplasmic trehalase that contributes to osmotic stress resistance was also shown to affect type 1 fimbriae production in an APEC strain and this mutation significantly reduced adherence to and invasion of epithelial cell and bladder colonization in a murine model of UTI (Pavanelo et al., 2018). Taken together, there is a body of evidence indicating that type 1 fimbriae expression is important for UPEC colonization of the urinary tract and that multiple factors, including adaptation to osmotic and oxidative stress, that influence expression of these fimbriae play a role in *E. coli* UTI pathogenesis.

Given the importance of type 1 fimbriae and control of its expression, in the present report we devised a means to randomly identify different genes involved in the regulation of type 1 fimbriae by using a *luxCDABE* reporter fusion and genetic screening of a transposon bank. We constructed a *fimS-lux* reporter fusion integrated on a single-copy at the chromosomal *att*Tn*7* of pyelonephritis strain CFT073 (Figure 1B). Integrating the *lux* reporter into the chromosome results in a reduced level of promoter expression compared to fusions on multi-copy vectors, but has the added advantage of stability of the signal during long term localization studies and more relevant comparison to regulatory effects on the native *fim* switch, as the single-copy fusion will not be as biased by titration of regulator proteins or recombinases that could occur with multi-copy fusion vectors. Moreover, the luciferase bioluminescence system may overcome some limitations of fluorescent reporters used in *in vivo* imaging, such us background signals associated with cellular autofluorescence, poor penetration of the excitation wavelength, slow turnover of the fluorescent protein, etc. (Riedel et al., 2007).

The *fimS-lux* fusion was shown to undergo a change in signal in accordance to changes in regulation of the *fimA* promoter observed under different pH conditions (Figure 2A), demonstrating its potential value at investigating environmental cues that could affect transcription of the *fim* gene cluster. A shift from pH 4.4 to a neutral pH 7.0 resulted in a significant increase of *fimS* expression. Furthermore, and in accordance with previous reports (Schwan et al., 2002; Schwan and Ding, 2017), the expression of *fimS* was increased when bacteria were cultured in LB (neutral pH) and decreased at lower pH (Figure 2A). The *in vitro* results with the *fim-lux* fusions were similar to previous studies (Schwan et al., 2002; Schwan and Ding, 2017), and helped validate the reporter system that we developed. Our *lux* fusion was highly sensitive with low background noise, which makes this reporter rapid enough to enable a delicate monitoring of quick response kinetics. Indeed, such vectors have also been developed for transposon-based systems with random integration of *lux* into the chromosomes of both Gram-negative and Gram-positive bacteria (de Lorenzo et al., 1990; Francis et al., 2001). However, these systems are based on random integration of the transposon into the chromosome, followed by selection of clones which retain viability but demonstrate high levels of *lux* expression. Hence, our *lux* system seems more efficient with single-copy integration of recombinant genes at the *att*Tn*7* site that does not require selective pressure and can be used in a variety of *Enterobacteriaceae*. This system provides a useful tool for studying promoter regulation without introducing unforeseen genetic changes that influence the behavior of the strain *in vitro* and *in vivo*. The application of the *lux* reporter in combination with transposon mutagenesis and high-throughput sequencing herein provided a novel and valid approach to identify specific genes and begin to dissect genetic pathways linked to expression of type 1 fimbriae.

Our transposon library screening resulted in the identification of numerous insertions that deregulated expression of type 1 fimbriae. In this study, we initially screened 5,904 transposon mutants by measuring the level of luminescence following growth on LB broth and phenotypic screening for production of type 1 fimbriae by using a yeast agglutination assay (Figure 3). Here, we searched for genes with a significant increase or decrease in luminescence levels from specific transposon mutants compared to the control strain (CFT073, *fimS-lux*). The insertion of transposons in specific clones were shown to repress or activate *lux* expression *fimS* and hence deregulate type 1 fimbriae production (Figure 3A). Using high throughput sequencing, we then mapped the Tn*10* insertion sites of these mutants (Supplemental Figure 3), leading to the identification of numerous genes that significantly altered type 1 fimbriae production (Table 2). In addition to known structural and regulatory genes, genes identified included those involved in biogenesis of type 1 fimbriae and other fimbriae, amino acid biosynthesis, membrane transport, chaperones involved in protein fate and genes that are currently of unknown function. Confirmation of the role of these genes in type 1 fimbriae expression via the construction and characterization of specific mutants will be required to more clearly determine their mechanisms of action in the regulation of expression type 1 fimbriae and potential roles in UTI pathogenesis. Several clones had insertions within genes with metabolic functions. One of these clones disrupted *tdcB* (catabolic threonine dehydratase). Many nutrient transport systems and genes related to carbohydrate metabolism have been reported to be involved in the virulence of ExPEC strains. For example, the periplasmic trehalase *treA* affects type 1 fimbriae production and virulence of APEC strain MT78 (Pavanelo et al., 2018). The metabolic *frz* operon has also been shown to link the metabolic capacity of ExPEC with expression of genes required for adherence to the bladder epithelium; the presence of the *frz* operon favors the ON orientation of the invertible type 1 fimbriae promoter (Rouquet et al., 2009). The two-component-system (TCS), KguSR, involved in the control of utilization of α-ketoglutarate, has also been shown to be important for UPEC fitness during UTI (Cai et al., 2013). Interestingly, this system was disrupted in our mutant bank. As such, the roles of this TCS in type 1 fimbriae expression in UPEC merit further investigation. Moreover, proteins involved in the transport and catabolism of sialic acid, xylose, arabinose, and the biosynthesis of arginine and serine are highly expressed in UPEC cultured in human urine (Alteri et al., 2009). It has also been reported that sialic acid regulates type 1 fimbriae by inhibiting FimB switching in UPEC (Sohanpal et al., 2004). Tn insertions also disrupted well-studied regulators, Lrp, a global regulator of genes involved in metabolic functions within *E. coli* including fimbriae production (Brinkman et al., 2003; Kelly et al., 2006) and *fimB* which regulates phase variable expression of the *fim* operon (Blomfield et al., 1997). It is intriguing to see a potential connection of iron acquisition mechanisms and type 1 fimbriae production as disruption of the aerobactin precursor *iucC* (de Lorenzo and Neilands, 1986) affected the fimbriae production. However, the specific mechanisms underlying how certain genes involved in metabolism influence the expression of type 1 fimbriae and to what extent this alone may influence UPEC pathogenesis during UTI remain to be elucidated.

Further, some of the genes in our bank have been reported earlier for the correlation with strong virulence and pathogenesis of UPEC. Loss of *nadB* rendered NAD auxotrophy and contributes to the virulence in *Shigella* (Prunier et al., 2007) however the colonization of CFT073 and UTI89 in murine UTI model was not influenced by NAD auxotrophy (Li et al., 2012). We identified an insertion in *rstA* which is a part of low pH sensing RstBA two- component system which is directly involved in the regulation of *csgD*, which encodes a regulator responsible for curli and cellulose production (Ogasawara et al., 2010). Interestingly, mutation in *emrK* in *E. coli* caused a significant drop in biofilm formation (Matsumura et al., 2011). So, these observations show the possible correlation between inhibition of fimbriae and a reduction in biofilm formation.

This relation has been documented before in terms of aerobic respiration in the bladder. Biofilm production in UPEC has been shown to be affected by different terminal electron acceptors (Eberly et al., 2017). In addition, type 1 fimbriae production was reduced in the absence of oxygen, and UPEC strain UTI89 had increased type 1 fimbriae production on the air exposed region of biofilm due to increased oxygen level (Floyd et al., 2015). In conjugation with biofilm forming capacity, cytochrome *bd* provided a fitness advantage for UTI89 under hypoxic growth conditions as well as increased nitric oxide tolerance (Beebout et al., 2019). So, our insertion in cytochrome c-type gene, *yecK/torY*, may also affect the adherence due to type 1 fimbriae as well as respiration in various oxygen gradient in the complex biofilm community.

Interestingly, many of the genes identified in our bank that influenced type 1 fimbriae expression are uncharacterized genes of hypothetical or unknown function. We were particularly interested in further investigating the role of the *yqhG* gene encoding a hypothetical protein containing DUF3828 domains that is conserved in *E. coli*. Although, the function of this gene is still unknown, transcription of *yqhG* was shown to be positively regulated by the BglJ-RcsB complex (Salscheider et al., 2013) in *E. coli* K-12. A putative binding site for BglJ-RcsB with a significant score was also identified upstream of the *yqhG* promoter (Salscheider et al., 2013). Interestingly, RcsB is the response regulator of the Rcs phosphorelay which is conserved in *Enterobacteriaceae*. RcsB plays a pleiotropic role in the control of biofilm formation, motility behavior and responds to membrane stress, specifically outer membrane stress, and is best known for its positive regulatory effect on capsule synthesis (Majdalani and Gottesman, 2005; Majdalani et al., 2005). Further, BglJ is a positive DNA-binding transcriptional regulator of transport and utilization of the aromatic β-glucosides arbutin and salicin (Madhusudan et al., 2005). It has been shown that it is completely dependent on RcsB (Venkatesh et al., 2010). It will therefore be of interest to further investigate potential regulatory links between these regulators and *yqhG* expression in UPEC.

A more pronounced reduction of yeast agglutination occurred when the *yqhG* mutant was grown to mid-log phase in LB or urine compared to O/N growth. Similarly, other mutations in genes, such as the *pst* system also resulted in a marked change in expression of type 1 fimbriae during growth to mid-log phase compared to after overnight static growth (Crépin et al., 2012b). Because the CFT073 Δ*yqhG* mutant displayed a decreased capacity to agglutinate yeast in LB and urine (Figures 4B,C), we performed Western blotting against type 1 fimbriae to investigate any effect on type 1 fimbriae production. As shown in Figure 4D in comparison to the WT strain, the mutant had a reduced production of type 1 fimbriae. This reduction in type 1 fimbriae was also in line an increased bias for the OFF position with *fim* promoter in the *yqhG* mutant in LB broth at O.D._600nm_ 0.6 (Figure 5B). Interestingly, we also found that the *yqhG* mutant was more motile than the WT strain (Figure 7). UPEC strains coordinately regulate motility and adherence to mediate colonization and dissemination during the pathogenesis of UTIs. It is widely believed that when adhesin genes are expressed, motility genes are repressed and *vice versa* (Simms and Mobley, 2008). They represent opposing forces. Thus, by mediating adherence, fimbriae would promote a sessile state and flagellar-based motility would be expected to be decreased. By contrast, increased motility by flagella would reduce the ability of bacteria to adhere at one site. Accordingly Bryan et al. (2006) and Lane et al. (2007) have shown that constitutive expression of type 1 fimbriae (CFT073 *fim* L-ON) leads to repression of swimming motility in strain CFT073. The *yqhG* mutant also exhibited increased binding to Congo Red on agar plates, suggesting a possible increase in cellulose and curli production compared to the wild-type strain. The decreased expression of type 1 fimbriae may therefore also lead to increased production of other adhesins or biofilm associated factors under certain growth conditions. Further experiments, will be required to confirm whether loss of *yqhG* may promote expression of other fimbriae while reducing expression of type 1 fimbriae.

In the murine UTI model, the *yqhG* mutant was attenuated (Figure 5). Loss of *yghG* in CFT073 caused an important decrease in colonization of both the bladder and kidneys, whereas loss of type 1 fimbriae by deletion of the *fim* operon, mainly results in a decrease in colonization of the bladder in the mouse model (Gunther et al., 2002). As such, we investigated whether loss of *yqhG* also affected resistance to certain stress conditions including oxidative stress and osmolarity. A Δ*yqhG* derivative of CFT073 was significantly more sensitive than its wild-type parent to oxidative stress from H_2_O_2_ challenge (Table 3), and complementation of this mutant with a single-copy of *yqhG* restored wild-type resistance to H_2_O_2_ killing. Although, it is clear that YqhG plays a role in regulating expression of type 1 fimbriae and promoting adherence of UPEC CFT073 to host cells, YqhG likely plays a greater role in UPEC, including adaptation to environmental stresses, as it is also required for resistance to oxidative stress. A plausible mechanism for this drop could be due to reduced catalase activity in the mutant to counteract the oxidative burst from immune cells during infection. The *yghG* mutant colonies also demonstrated less bubbling upon addition of H_2_O_2_ compared to wild type. So, mutation in *yqhG* may also lead to increased sensitivity to reactive oxygen radicals as well as reduced production of type 1 fimbriae. Nevertheless, our results shed new light on the importance of *yqhG* for UPEC virulence, and it will be of interest to further elucidate what other factors that are, directly or indirectly, regulated by *yqhG*, including type 1 fimbriae, and determine its importance for resistance of *E. coli* to host innate immune response during infection.

Finally, we investigated whether inactivation of *pst*, in addition to repression of expression of type 1 fimbriae also reduced resistance to stresses including oxidative and osmotic stress. Decreased virulence of the *pst* mutant of UPEC CFT073 was mainly attributed to the decreased expression of type 1 fimbriae (Crépin et al., 2012b). The ability sense, adapt to, and resist different types of stress can also play an important role in regulation of gene expression, including regulation of type 1 fimbriae. As such, we investigated the capacity of the *pst* mutant to resist oxidative and osmotic stresses. The conditions were chosen to simulate the effects of NaCl and urea that UPEC cells may encounter during colonization of the urinary tract. Urea can permeate through the cell membrane and destabilize the native structure of proteins inside cells (Withman et al., 2013). According to our results, CFT073 showed a higher resistance to 0.6 M urea and 0.6 NaCl than the *pst* mutant grown to mid-log phase (Figures 7C,D). Similarly, the *pst* mutant was much more sensitive to hydrogen peroxide than the CFT073 parent strain (Table 3). Therefore, inactivation of *pst* in CFT073 resulted in increased sensitivity to both osmotic and oxidative stress, and this may importantly also in part be linked to changes in levels of expression of type 1 fimbriae. In APEC strain χ7122, inactivation of the Pst system induced deregulation of phosphate sensing and important changes in cell surface composition that led to reduced virulence in a chicken infection model, decreased production of type 1 fimbriae and lower resistance to oxidative stress (Lamarche et al., 2005; Crépin et al., 2008). Several studies concerning osmotic stress and expression of type 1 fimbriae were also reported for UPEC. In UPEC strain NU149, type 1 fimbriae expression was downregulated under osmotic stress caused by NaCl (Schwan et al., 2002). Further, loss of *treA* in ExPEC strain MT78 also resulted in a change in osmotic resistance to urea, concomitant with a decreased expression of type 1 fimbriae and reduced urinary tract colonization in the mouse model (Pavanelo et al., 2018). Transcriptome analyses also showed upregulation of type 1 fimbriae expression and the genes that are regulated by the osmotic stress response in CFT073 during UTI (Snyder et al., 2004).

Overall, generation of a transposon bank and a single-copy *lux*-based reporter fusion system integrated at the *att*Tn7 site in UPEC strain CFT073, has led to successful identification of insertions in a number of genes including heretofore unknown sites that altered expression of type 1 fimbriae. Interestingly, these insertions include a variety of genes involved in a diversity of functions including protein fate and synthesis, energy metabolism, adherence, transcriptional regulation, and transport, and genes of hypothetical or unknown function including *yqhG*, that we have shown to play an important role in UPEC colonization in the mouse model as well as resistance to oxidative stress. It will be of interest to more fully elucidate how some of these different systems influence expression of type 1 fimbriae as well as their potential roles in metabolism and bacterial regulatory networks as well as sensing, adaptation and resistance to environmental stresses, such as osmotic and oxidative stresses that may be encountered during the course of colonization and infection of the host.

## Data Availability

The datasets generated for this study are available on request to the corresponding author.

## Author Contributions

HB was the primary author and performed most of the experiments and writing of the manuscript. PP and HH contributed to some of the experiments and writing of the text. SH contributed the technical assistance including mouse infections with other co-authors. CD conceived the planning of the study, design of experiments, mentored the researchers, and revised the manuscript.

### Conflict of Interest Statement

The authors declare that the research was conducted in the absence of any commercial or financial relationships that could be construed as a potential conflict of interest.

## Supplementary Material

Supplemental Figure 1 Production of type 1 fimbriae by uropathogenic *E. coli* CFT073 and its derivative strains. **(A)** Production of type 1 fimbriae in strains cultured to the mid-log phase of growth in LB broth in different pH **(B)** O/N culture **(C)** in LB plate. The CFT073 *fim-*LON strain was used as a positive control for agglutination. Results are the mean values and standard deviations for three biological experiments. Statistical significance was calculated by the one-way ANOVA. ^*^*P* < 0.05; ^**^*P* < 0.005; ^***^*P* < 0.0001.

Supplemental Figure 2 **(A)** Growth curve optical density and **(B)** viable counts (CFU/ml) of wild type CFT073 and its P*fimA-*Locked ON and P*fimA* variable *lux* derivatives. The cells were grown in LB broth. There was no significant differences in the growth phases of all strains.

Supplemental Figure 3 Transposon mutants with affected expression of type 1 fimbriae in CFT073. Genetic locus with the closest match to the sequence interrupted by the transposon in each mutant are represented with an arrow (teal) in their respective insertion site.

Supplemental Table 1 Primers used in this study.
