# Supplementary figures and images for: yqhG Contributes to Oxidative Stress Resistance and Virulence of Uropathogenic Escherichia coli and Identification of Other Genes Altering Expression of Type 1 Fimbriae
Source: Front Cell Infect Microbiol. 2019 Aug 29;9:312. doi: 10.3389/fcimb.2019.00312 (PMC6727828; doi:10.3389/fcimb.2019.00312)

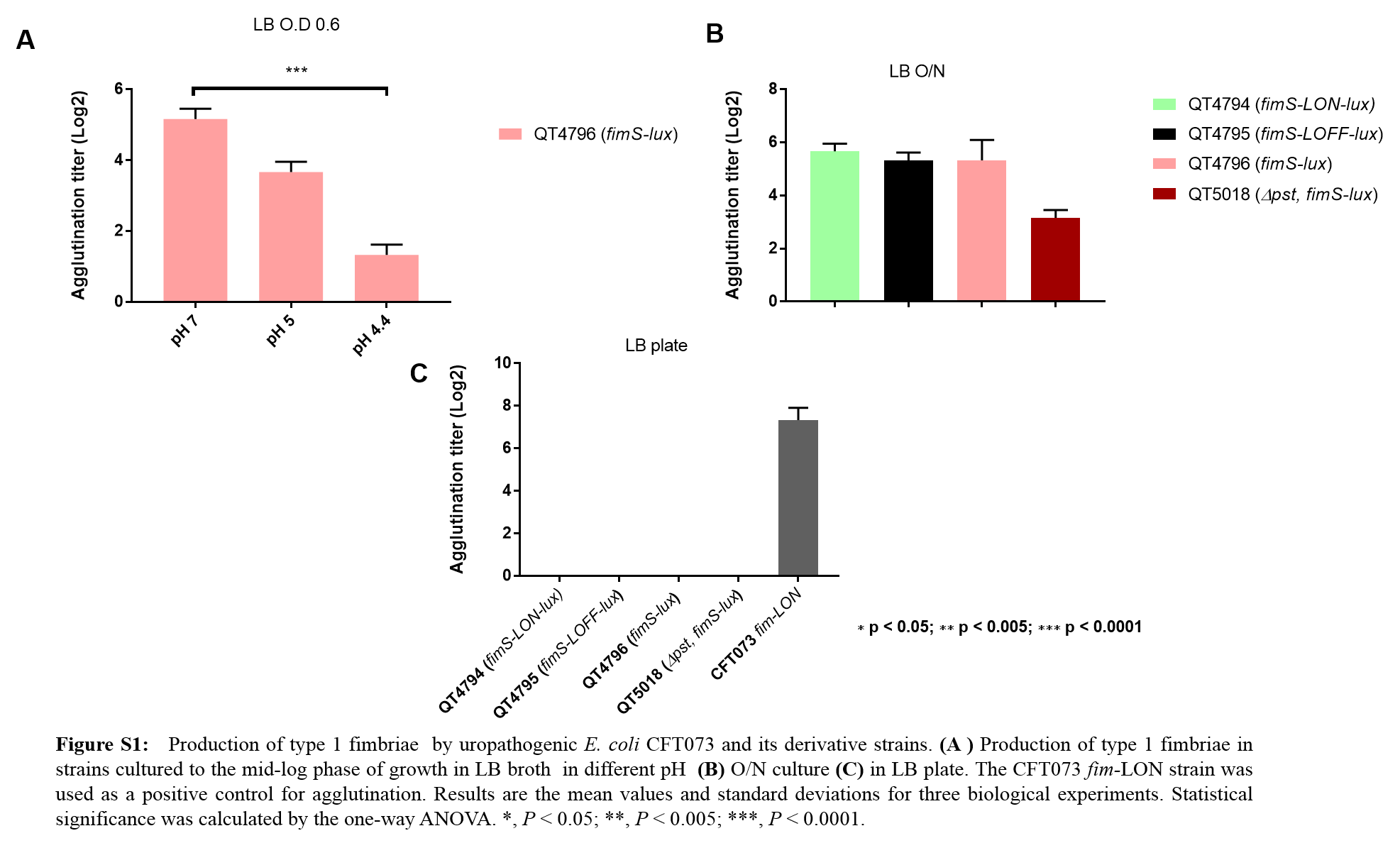

Supplement: Supplemental Figure 1 — Production of type 1 fimbriae by uropathogenic E. coli CFT073 and its derivative strains. (A) Production of type 1 fimbriae in strains cultured to the mid-log phase of growth in LB broth in different pH (B) O/N culture (C) in LB plate. The CFT073 fim-LON strain was used as a positive control for agglutination. Results are the mean values and standard deviations for three biological experiments. Statistical significance was calculated by the one-way ANOVA. *P < 0.05; **P < 0.005; ***P < 0.0001. [file Image_1.tif]

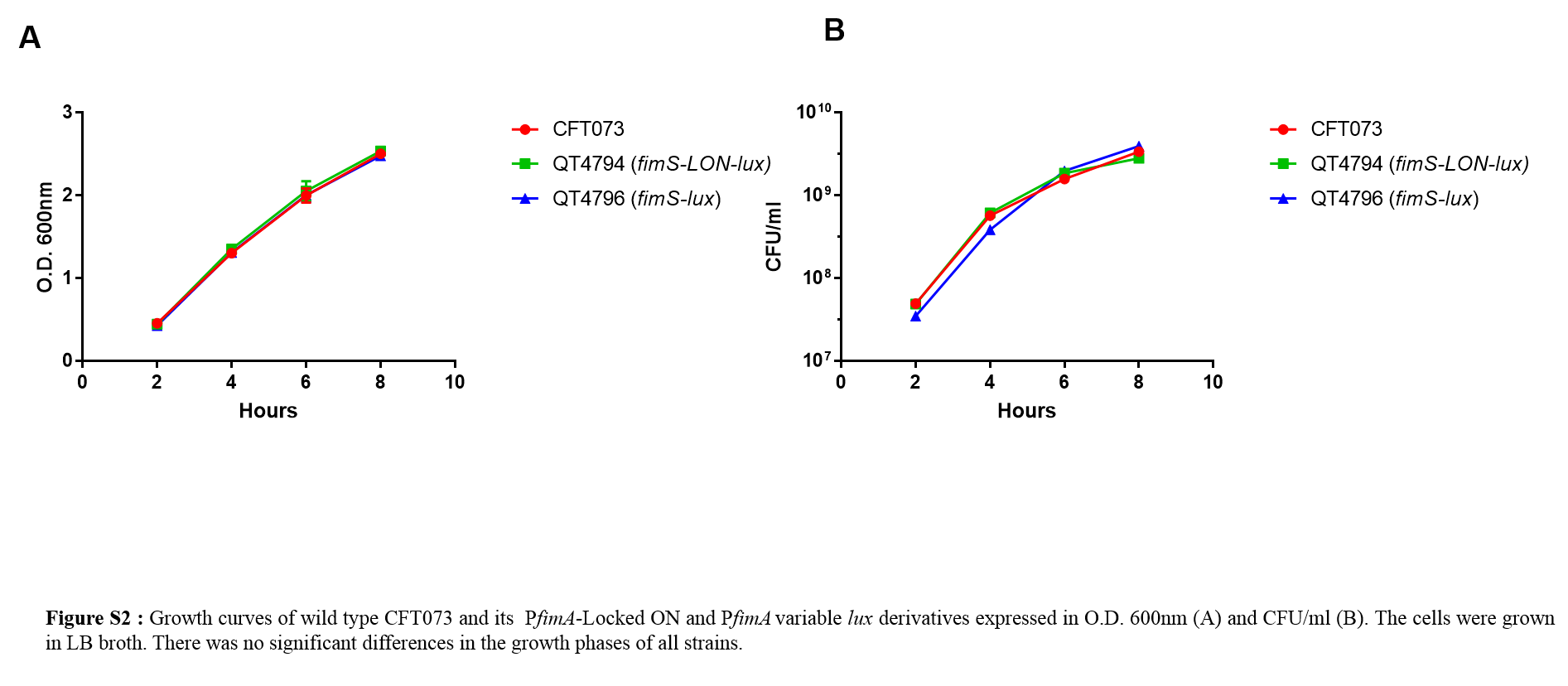

Supplement: Supplemental Figure 2 — (A) Growth curve optical density and (B) viable counts (CFU/ml) of wild type CFT073 and its PfimA-Locked ON and PfimA variable lux derivatives. The cells were grown in LB broth. There was no significant differences in the growth phases of all strains. [file Image_2.tif]

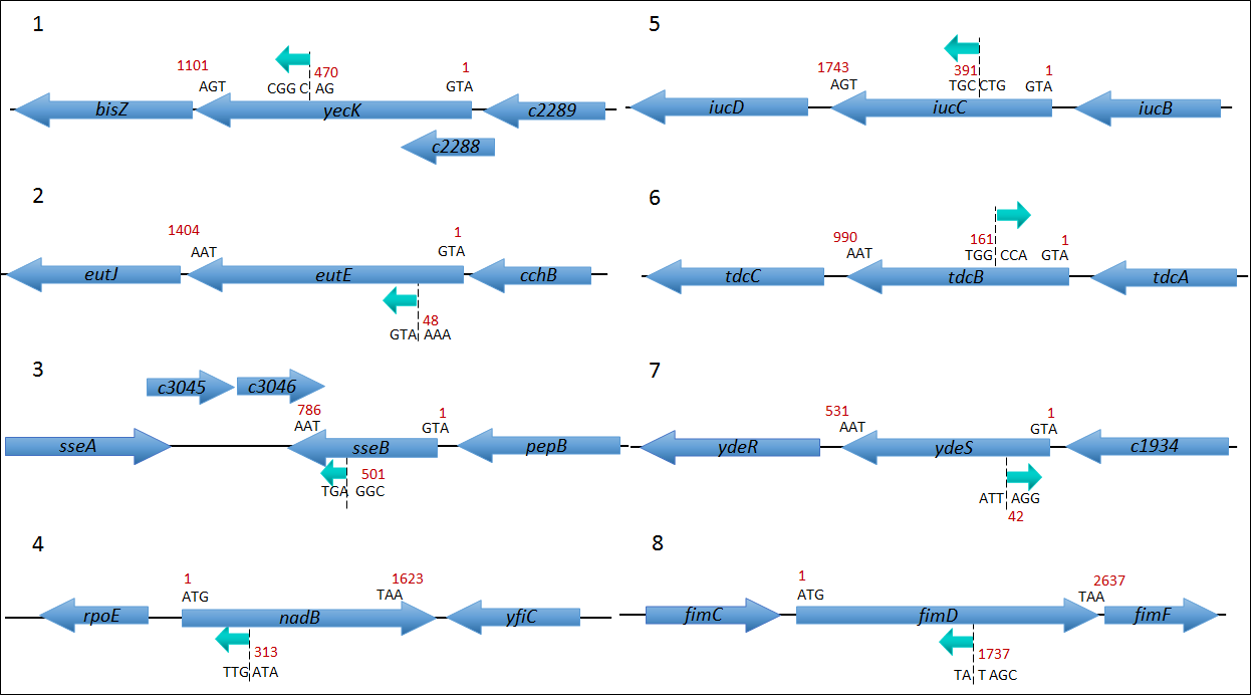

Supplement: Supplemental Figure 3 — Transposon mutants with affected expression of type 1 fimbriae in CFT073. Genetic locus with the closest match to the sequence interrupted by the transposon in each mutant are represented with an arrow (teal) in their respective insertion site. [file Image_3.TIF]

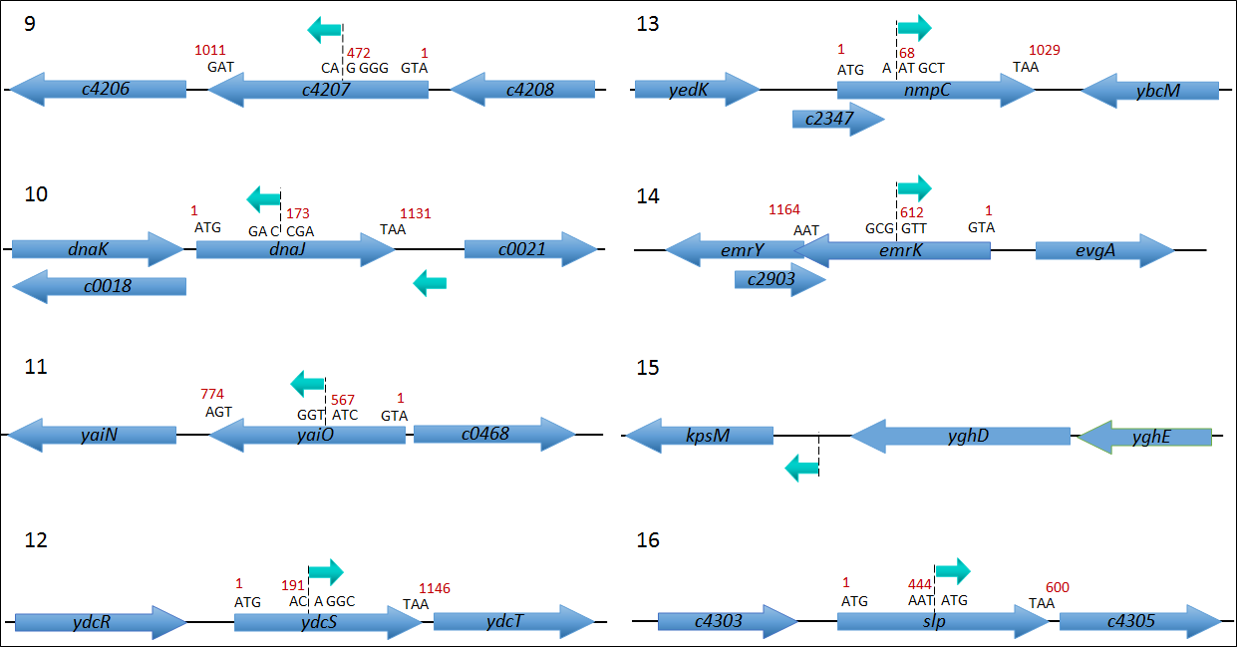

Supplement: Supplementary file 5 [file Image_4.TIF]

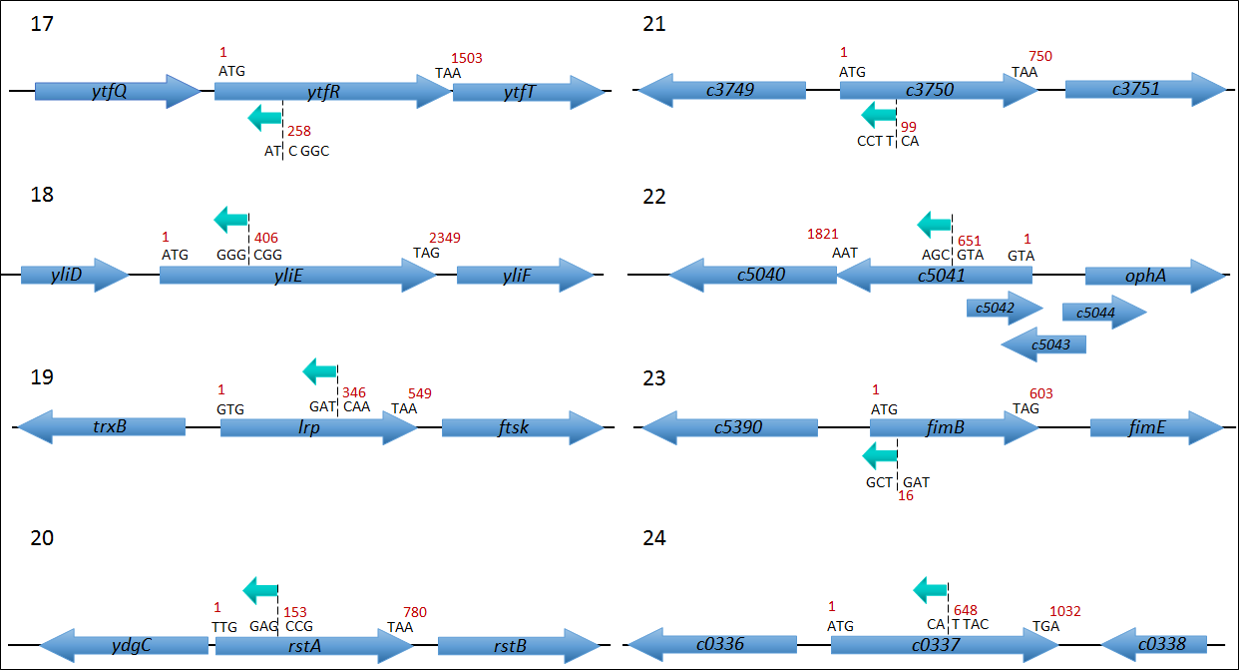

Supplement: Supplementary file 6 [file Image_5.TIF]

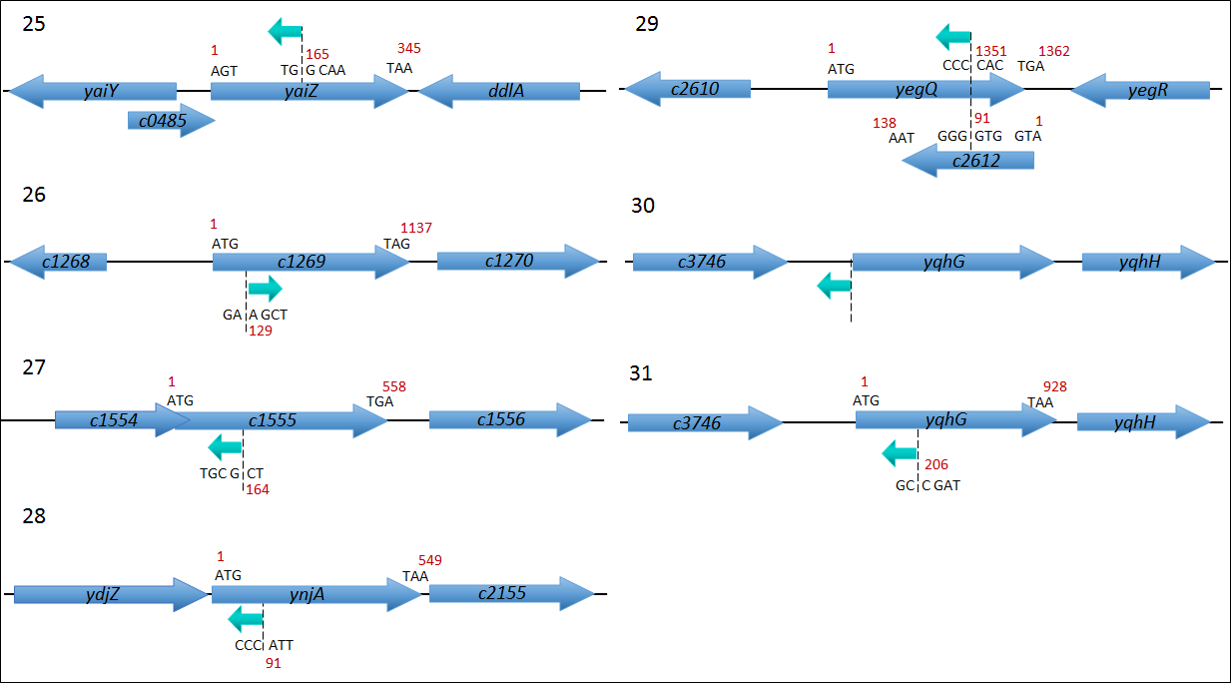

Supplement: Supplementary file 7 [file Image_6.TIF]
