# Supplementary material for: yqhG Contributes to Oxidative Stress Resistance and Virulence of Uropathogenic Escherichia coli and Identification of Other Genes Altering Expression of Type 1 Fimbriae
Source: Front Cell Infect Microbiol. 2019 Aug 29;9:312. doi: 10.3389/fcimb.2019.00312 (PMC6727828; doi:10.3389/fcimb.2019.00312)
Supplement: Supplemental Table 1 — Primers used in this study. [file Table_1.DOCX]

Supplemental Table 1. Primers used in this study

| Primers | Direction | Characteristic(s) | Sequences 5' ➝ 3' |
| --- | --- | --- | --- |
| CMD26 | Forward | In *glmS* for screening integration in *att*Tn7 site (used with CMD1416) | GAT CTT CTA CAC CGT TCC GC |
| CMD1133 | Forward | Amplification of *fimS* promoter phase locked ON used with CMD1646) | ACA ATG CCC GGG CTT TTG ACT CAT AGA GGA AAG CAT C |
| CMD1186 | Reverse | Amplification of ON-orientation of *fimS* (used with CMD1185 | **^Ψ^**AAAGTTTGTGCGCGATGCTTTCCTCTATGAGTCAAAATAGATCTCCTTGATGGGAATTAGCCATGGTCC |
| CMD1185 | Forward |  | TGGGAAAGAAATAATCTCATAAACGAAAAATTAAAAAGAGAAGAAGTTTGGTGTAGGCTGGAGCTGCTTC |
| CMD1246 | Forward | Amplification of a fimE-IE region (used with CMD1248) | GAT CTT CTA CACCGT TCC GC |
| CMD1247 | Forward | Amplification of a IE-fimA region (used with CMD1248) | TGAACGGTCCCACCTTAACCG |
| CMD1248 | Reverse |  | TCACATCACCCCGCTATATGT |
| CMD1258 | Forward | Amplification of the fimS region (used with CMD1259) | TCGTTTTGCCGGATTATGGG |
| CMD1259 | Reverse |  | AGTGAACGGTCCCACCATTAACC |
| CMD1416 | Reverse | In Tn7 (Used with CMD26) | GCT TTT TCA CAGCAT AAC TGG A |
| CMD1645 | Forward | Amplification of *fimS* (used with CMD1646) | TTT AAC GAA TTC ATA ATA AAG TTA AAA AAC AAA TAA ATA CAA GAC |
| CMD1646 | Reverse |  | CAT GTC GAC TTC CTT TAA AAA AAC TAT TTC TAA ATC GAC ATG GGC |
| CMD1733 | Forward | Amplification of the *luxCDABE* operon without promoter (used with CMD 1734) | CGG TAC CCT AAC TAT CAA ACG CTT CGG TTA AGC TTA AAG CAC |
| CMD1734 | Reverse |  | CGG TAC CCTAACTAT CAA ACGCTT CGGTTA AGC TTAAAGCAC |
| CMD2290 | Forward | Amplification of the *Km* gene from QT5134 (*yqhG::km*) (used with CMD2291) | \| TCATCATGCAGAGCCGGAAG \| \| --- \| \|  \| |
| CMD2291 | Reverse |  | \| CAGGTTGCCAGAACAACAGC \| \| --- \| |
| CMD2318 | Forward | Cloning of *yqhGH* into pGP-Tn7-Cm plasmid and amplification of *yqhGH* fragment for complementation (used with CMD2320) | GCAAGGCCTTCGCGAGGTACCACTCTTCCTTGTTCAGGAAT |
| CMD2320 | Reverse |  | CGGGCTGCAGGAATTCCTCGAG ATATCCCCGGTAACAGAATG |

**^Ψ^**The red underlined nucleotides of the CMD 1186 primer were modified from the original sequence to lock the promoter switch (*fimS*) to the ON (L-ON) position.
